# A Combined Bioassay and Nanofractionation Approach to Investigate the Anticoagulant Toxins of Mamba and Cobra Venoms and Their Inhibition by Varespladib

**DOI:** 10.3390/toxins14110736

**Published:** 2022-10-27

**Authors:** Arif Arrahman, Taline D. Kazandjian, Kristina B. M. Still, Julien Slagboom, Govert W. Somsen, Freek J. Vonk, Nicholas R. Casewell, Jeroen Kool

**Affiliations:** 1 Division of Bioanalytical Chemistry, Department of Chemistry and Pharmaceutical Sciences, Faculty of Sciences, Amsterdam Institute of Molecular and Life Sciences (AIMMS), Vrije Universiteit Amsterdam, De Boelelaan 1083, 1081 HV Amsterdam, The Netherlands; 2 Centre for Analytical Sciences Amsterdam (CASA), 1012 WX Amsterdam, The Netherlands; 3 Faculty of Pharmacy, Universitas Indonesia, Kampus Baru UI, Depok 16424, Indonesia; 4 Centre for Snakebite Research and Interventions. Liverpool School of Tropical Medicine, Pembroke Place, Liverpool L3 5QA, UK; 5 Naturalis Biodiversity Centre, Darwinweg 2, 2333 CR Leiden, The Netherlands

**Keywords:** snakebite, coagulopathy, varespladib, marimastat, *Dendroaspis*, *Naja*, mass spectrometry

## Abstract

Envenomation by elapid snakes primarily results in neurotoxic symptoms and, consequently, are the primary focus of therapeutic research concerning such venoms. However, mounting evidence suggests these venoms can additionally cause coagulopathic symptoms, as demonstrated by some Asian elapids and African spitting cobras. This study sought to investigate the coagulopathic potential of venoms from medically important elapids of the genera *Naja* (true cobras), *Hemachatus* (rinkhals), and *Dendroaspis* (mambas). Crude venoms were bioassayed for coagulant effects using a plasma coagulation assay before RPLC/MS was used to separate and identify venom toxins in parallel with a nanofractionation module. Subsequently, coagulation bioassays were performed on the nanofractionated toxins, along with in-solution tryptic digestion and proteomics analysis. These experiments were then repeated on both crude venoms and on the nanofractionated venom toxins with the addition of either the phospholipase A_2_ (PLA_2_) inhibitor varespladib or the snake venom metalloproteinase (SVMP) inhibitor marimastat. Our results demonstrate that various African elapid venoms have an anticoagulant effect, and that this activity is significantly reduced for cobra venoms by the addition of varespladib, though this inhibitor had no effect against anticoagulation caused by mamba venoms. Marimastat showed limited capacity to reduce anticoagulation in elapids, affecting only *N. haje* and *H. haemachatus* venom at higher doses. Proteomic analysis of nanofractionated toxins revealed that the anticoagulant toxins in cobra venoms were both acidic and basic PLA_2_s, while the causative toxins in mamba venoms remain uncertain. This implies that while PLA_2_ inhibitors such as varespladib and metalloproteinase inhibitors such as marimastat are viable candidates for novel snakebite treatments, they are not likely to be effective against mamba envenomings.

## 1. Introduction

Snakebite is a neglected tropical disease, affecting between 421,000 [1] and 2.7 million individuals [2] each year and resulting in 81,000–138,000 deaths. Estimates of global envenoming rates fluctuate dramatically due to the challenges of collecting consistent and reliable data, which relies on the reporting of snakebite incidences and mortality from both individuals in the community and hospitals [3]. The effects of snakebite are most pronounced in low and middle-income regions [4]. This is due to a combination of factors such as lack of antivenom availability, limitations relating to healthcare infrastructure, accessibly and appropriately trained medical staff, and the ability to afford effective treatment and rehabilitation. Such factors have led to an estimated 93 million people living in locations classed as being vulnerable to snakebite [5], culminating in snakebite being listed by the World Health Organization as a priority neglected tropical disease.

One of the most medically important groups of snakes are the elapids (Serpentes: Elapidae), a snake family that consists of over 300 species, of which the World Health Organization considers around 80 to have medical importance in the context of human snakebite [2,6]. Elapid venoms are complex mixtures of pharmacologically active proteins and polypeptides that play an essential role in the incapacitation, immobilisation, and digestion of prey [7,8,9]. These venoms tend to be dominated by the presence of three-finger toxins (3FTXs) and/or phospholipase A_2_ (PLA_2_) toxins [6,10], which often act upon the neuromuscular junction to cause paralysis and respiratory failure [11]. Arguably the most medically important elapids in sub-Saharan Africa are the cobras and near relatives (genus *Naja* and *Hemachatus haemachatus*) and the mambas (genus *Dendroaspis*) [12]. While both groups of these elapid snakes employ a subfamily of venom 3FTXs known as α-neurotoxins to incapacitate prey, cobra venoms also contain a large abundance of cytotoxic 3FTX which, in the context of spitting cobras at least, likely contribute to causing pain and cell damage [13,14], while mamba venoms contain large amounts of dendrotoxins [15], which contribute to neurotoxic symptoms via the blockage of potassium channels [16,17].

Though the predominant symptom of elapid envenoming is neurotoxicity, there is evidence that some of these venoms can additionally display hemotoxic effects. For example, some Australian elapid snakes have venoms that cause potent procoagulation by activating prothrombin [18,19,20,21]. However, certain elapid venoms have anticoagulant effects, which are achieved through several mechanisms: for example, the inhibition of factor X or thrombin, the lysis of fibrin, the activation of plasminogen and the inhibition of platelet aggregation [22,23,24,25,26,27]. Rather than acting directly to incapacitate prey, as observed for the procoagulant toxins of some Australian species, toxins that prevent coagulation could potentially aid in the spread of neurotoxins throughout the body leading to quicker systemic envenomation, though the pathological relevance of such effects in human envenomings remains unclear. Anticoagulant activity has been reported in the venoms of several cobra species [25,28,29]. Cobras contain several venom toxins with the potential for exerting anticoagulant activity, including PLA_2_s, which can act to inhibit platelet aggregation [30], thrombin [31,32], the tenase and prothrombinase complexes [33,34], and clotting factor Xa (FXa) [25] and 3FTXs, of which cytotoxic 3FTXs (CTXs) have been shown to lyse erythrocytes [35,36,37] and, in the venom of *Hemachatus haemachatus*, form a complex called hemextin A & B that inhibits the activity of the coagulation factor VIIa. However, recent studies have identified PLA_2_s as the likely causative agents, both via direct identification following toxin fractionation for *Naja nigricollis* [38], and via functional inhibition of PLA_2_ toxins for a much broader range of spitting cobra species [39]. However, there is a lack of such studies focusing on mamba venoms, though prior research suggests they might inhibit platelet aggregation [40].

Current snakebite treatments, known as antivenom and which consist of animal-derived polyclonal antibodies stimulated by snake venoms, are delivered intravenously to snakebite victims. There are several commercially available antivenoms produced for the treatment of cobra and mamba bites in India and Sub-Saharan Africa [41,42,43,44,45], produced using either a single species (monovalent) or covering a range of species (polyvalent). While these are life-saving therapies, they have several limitations associated with their use, including extremely high costs to consumers, limited cross-species efficacy [43,45], cold storage requirements, high incidences of adverse reactions, and a requirement to be delivered intravenously in a healthcare setting [46,47,48,49,50,51]. Additionally, there is a lack of preclinical testing for current antivenoms, whose production methods differ very little today than when the first antivenoms were produced, and the existing preclinical data are not standardised and often not publicly available [45]. Thus, research into refining antivenoms, or finding treatments that can be used synergistically with antivenoms to improve patient outcomes further, are a priority in the field.

One avenue in the pursuit of this goal is research into small molecule inhibitor s [52,53]. Varespladib [54] and marimastat [55] are small molecule drugs that inhibit the activity of PLA_2_s and matrix metalloproteases, respectively. Originally intended as treatments for inflammatory diseases and cancer, they have since been explored for repurposing as snakebite treatments. Varespladib in particular is a broad-spectrum inhibitor of venom PLA_2_s that has been shown to delay the lethal effects of venoms from the elapids *Bungarus multicinctus*, *Oxuranus scutellatus*, and *Micrurus fulvius* in mice through the inhibition of presynaptically acting PLA_2_s [56,57,58], with the injectable form temporarily reversing paralytic effects observed from the prior two species [57]. Furthermore, it confers a significant survival benefit when administered to mice envenomed by lethal doses of venom from certain vipers [59,60,61] and elapids [61], and provides protection against the pathologies of *Micrurus dumerilii carinicauda* venom in rats when used in combination with antivenom [62]. In terms of its ability to treat haemotoxic pathologies, in vitro studies demonstrate the ability of varespladib to neutralise the anticoagulant activity of PLA_2_s isolated from the venoms of the vipers *Echis carinatus* and *E. ocellatus*, and the elapid *Oxuranus scutellatus*, and it additionally exhibits some neutralisation of the procoagulant activity of PLA_2_s from several viper species [63]. Additionally, it has been shown to neutralise PLA_2_-mediated prothrombinase inhibition in whole venoms from the viper genus *Bitis* [64] and elapid genus *Pseudechis* [39], as well as negate [63] the anticoagulant activity of several spitting cobra species [25,38,39], which could not be neutralised by antivenom alone [25,57]. Similarly, marimastat has been found to inhibit the activity of viper SVMP toxins and is capable of conferring preclinical protection against hemotoxic pathology caused by certain envenomings in small animal models, especially when combined with antivenom [53,65,66,67,68,69]. It also prevents haemorrhage in mice from venoms of the vipers *Deinagkistrodon acutus* and *Agkistrodon halys* [70], inhibits the fibrinogenolytic activity of CAMP-2 from Crotalus atrox venom [67], and neutralises the SVMP-mediated procoagulant effects of venom from the colubrids *Rhabdophis tigrinus* [67] and *Dispholidus typus* [68]. These studies provide enticing evidence to pursue small molecule drugs as (at least adjuvant) treatments for snakebite envenomation.

**Figure 1:**
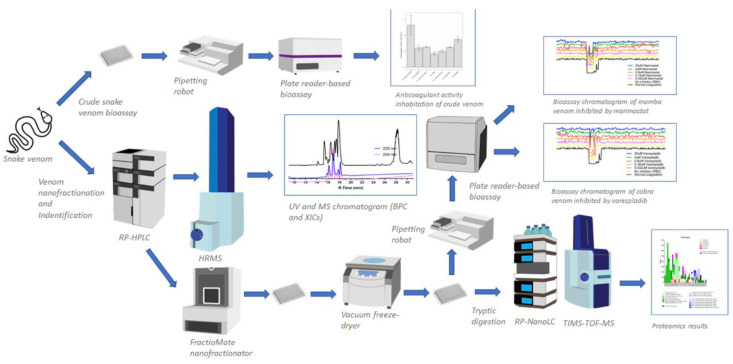
A schematic overview of the complete analytical and biochemical workflow. There are two main experiments shown in Figure 1 that run simultaneously in this study: (i) crude snake venom plate reader-based bioassaying (for assessing inhibition potential of anticoagulant activity by the small molecule inhibitors varespladib and marimastat), (ii) venom separation (using reversed-phase high-performance liquid chromatography; RP-HPLC or LC in short) coupled to UV detection followed by mass spectrometry (MS) with parallel nanofractionation for high-resolution fraction collection of separated toxins onto 384-well plates. Well plates with nanofractionated venom toxins are then vacuum centrifuged to dryness overnight, followed by bioassaying to assess anticoagulant activity. This data obtained is then processed to deliver bioassay chromatograms. Dried well plates can also be used for subsequent toxin identification by proteomics. Key: TIMS-TOF-MS, trapped ion mobility spectrometry time of flight mass spectrometry; HRMS, high-resolution mass spectrometry (HRMS); RP-nanoLC, reversed-phase nanoflow liquid chromatography.

This study investigates the anticoagulant activity of venoms sourced from medically important cobra and mamba species on bovine plasma and seeks to identify the toxins responsible for such activity. In addition, we evaluated the role of the small molecule inhibitors varespladib and marimastat as potential inhibitors of the anticoagulant toxins found in the elapid venoms under study. The analytical approach involved a workflow consisting of three main steps. The first step was an initial screening of coagulopathic activity of the various crude venoms and then investigating the effect of the small molecules varespladib and marimastat on inhibiting the resulting coagulopathic activity. The second step was the characterisation of nanofractionated toxins in the presence and absence of the same small molecule toxins inhibitors. The third step involved proteomics-based identification of the resulting coagulopathic venom toxins. A schematic overview of the complete analytical and biochemical workflow including workflow explanation is provided in Figure 1. The combination of these experimental approaches demonstrated that small molecule inhibitor varespladib can neutralise the potent anticoagulation effects often observed from cobra venoms.

## 2. Results

### 2.1. Initial Screening of Crude Venom Coagulopathic Activity and Inhibition by Varespladib and Marimastat

**Figure 2:**
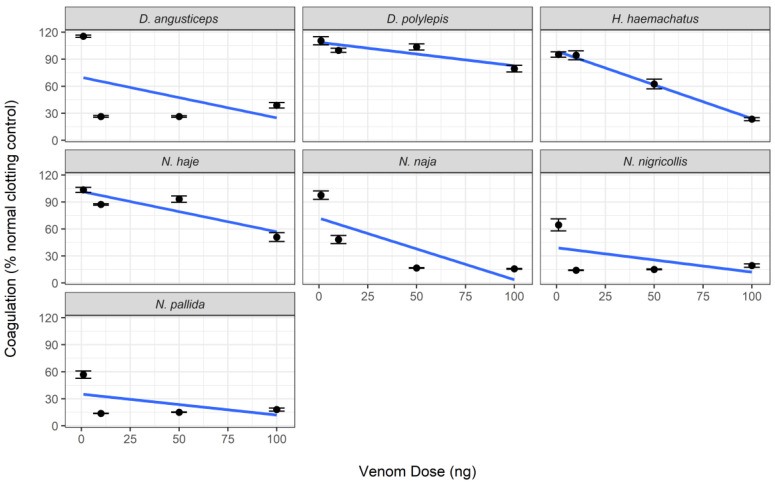
Venoms from elapids demonstrate anticoagulant activity on bovine plasma. Crude venom activity was measured as the mean area under the curve (AUC) of each venom concentration in the relevant clotting period (24–71 min), standardised by the mean AUC of the negative control in the same period. Lines were generated using a linear model and error bars represent the standard error of the mean (SEM) of 3–7 replicates (wells containing excess bubbles caused reading errors and had to be removed from analysis).

**Figure 3:**
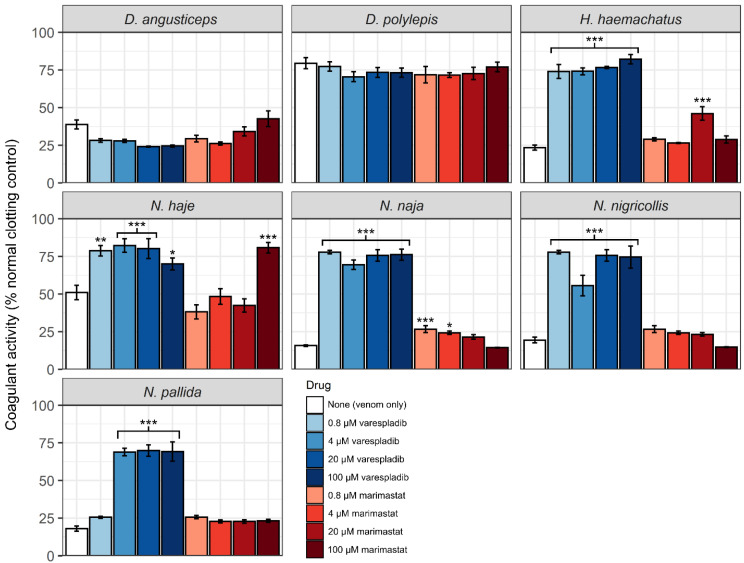
Anticoagulant venom activity of cobra venoms are inhibited by the PLA_2_ inhibitor varespladib. *H. haemachatus*, *N. haje*, and *N. naja* additionally show significant inhibition by some marimastat doses. Clotting activity was measured as the mean area under the curve (AUC) of each sample type in the relevant clotting period (22–71 min), standardised by the mean AUC of the negative control in the same period. Error bars represent the standard error of the mean (SEM) of 3–4 measurements (some sample wells needed to be removed due to disruption in readings caused by bubbles). Asterisks represent significant increases in coagulant activity as measured by one-way ANOVA and Dunnett’s Multiple Comparison post-hoc testing; *—*p* < 0.05, **—*p* < 0.01, ***—*p* < 0.0001.

Crude venom samples from elapids elicited an anticoagulant effect on bovine plasma, with venom from *Naja nigricollis* and *N. pallida* eliciting the strongest activity, and *Dendroaspis polylepis* eliciting the weakest activity (Figure 2 and Supplementary Figure S1). One-way ANOVA revealed significant reductions in anticoagulant activity in cobra venom with all doses of varespladib (*p* < 0.01), except in the case of *N. pallida*, which required a minimum dose of 4 µM to show significant reductions (*p* < 0.0001) (Figure 3, Supplementary Figures S2 and S3). This is in line with recently reported findings [39]. Varespladib caused no discernible effects on the coagulant activity of the mamba venoms. The effects of marimastat on the anticoagulant activity of elapid venoms are more difficult to disentangle. There is no significant effect of marimastat on mamba venoms, however *Hemachatus haemachatus, N. haje* and *N. naja* respond significantly to doses of 20 µM, 100 µM, and 4–20 µM, respectively (Supplementary Figures S2 and S4). The significant responses of *H. haemachatus* and *N. naja* may be statistical artifacts or errors, as it is unlikely that low doses of marimastat would stimulate a response but not higher doses; however the response of *N. haje* venom to 100 µM marimastat is comparable to that of varespladib (see Figure 2), suggesting that SVMPs do play a role in the anticoagulant activity of this venom, though seemingly a more minor one than that of PLA_2_s.

### 2.2. Nanofractionation, Identification, and Inhibition of Coagulopathic Mamba Venom Toxins

#### 2.2.1. *Dendroaspis polylepis*

**Figure 4:**
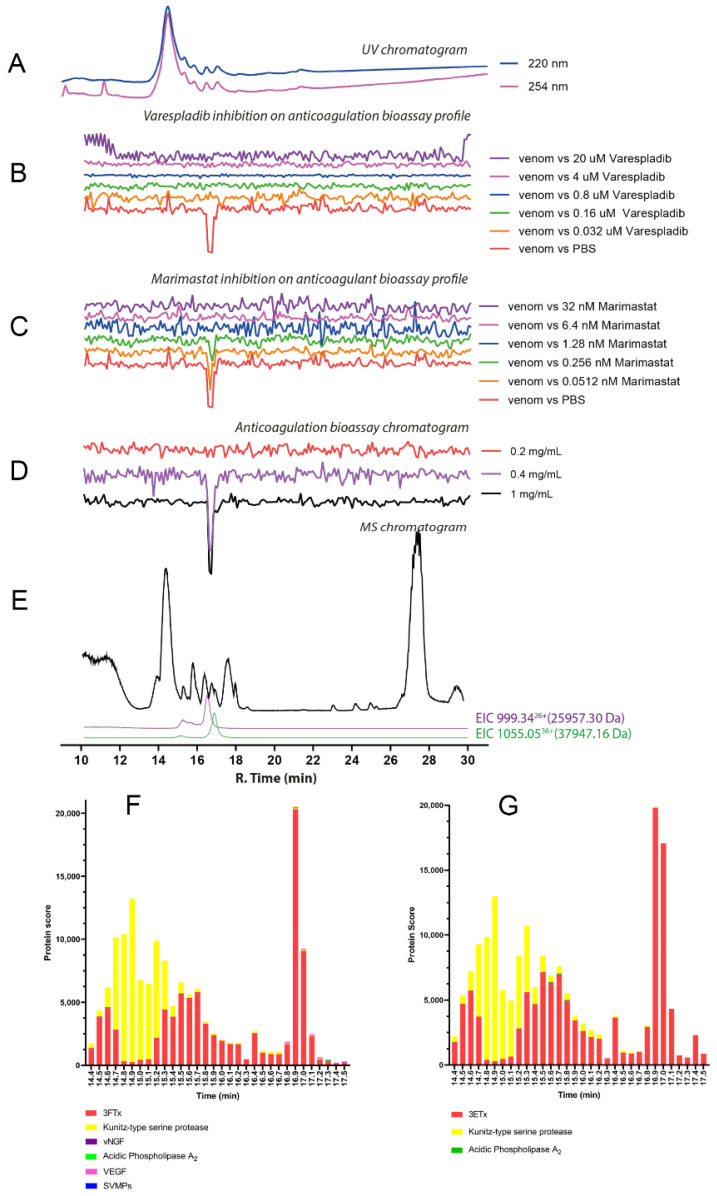
Correlation of LC-UV and MS chromatogram and bioassay chromatogram of *D. polylepis* venom. (**A**) UV chromatogram of *D. polylepis* venom (1 mg/mL, 50 μL injection volume, post-column split into 1:9 ratio. The smaller portion went to UV (220 and 254 nm recorded), and then to MS detection. Superimposed bioassay chromatograms resulting from analyses of *D. polylepis* venom (1 mg/mL, 50 µL injection volume) in the presence of different concentrations of (**B**) varespladib and (**C**) marimastat. (**D**) Superimposed anticoagulation bioassay chromatograms resulting from analyses of several diluted *D. polylepis* venom samples ranging from 1 mg/mL to 0.2 mg/mL (50 µL per injection). (**E**) Base peak chromatogram (BPC) and extracted ion currents (XIC) mass spectrometry. Bioassay chromatograms are correlated to UV and MS. Proteomic annotation of *D. polylepis* venom using Mascot software against (**F**) the Swiss-Prot database and (**G**) species-specific venom gland transcriptomic-derived database. The protein score represents the probability that designated proteins, defined by elution time, are present in the sample.

The chromatographic bioassay profile of *D. polylepis* venom showed a single sharp negative peak at a retention time of 16.5 min (Figure 4D). The lowest venom concentration analysed in which the anticoagulant toxin(s) was/were detected was 0.4 mg/mL (50 μL per injection, 20 μg of venom proteins), with further dilution (4 μg of venom proteins) rendering the anticoagulant activity undetectable. Noticeably, the extent of anticoagulant activity observed following nanofractionation was lower than anticipated based on findings with crude venom (Figure 4B,C). This may result from anticoagulant toxins present in *D. polylepis* venom (partially) denaturing during the reversed-phase LC separation. One molecular mass, at a retention time of 16.8 min, was assigned with an *m/z* of 1055.05 (charge +36; mol. mass 37,947.16 Da) for this active anticoagulant peak, falling within the snake venom metalloproteinases mass range (Figure 4E; Table 1). No protein was detected at the retention time of 16.8 min that corresponded to the PLA_2_ mass range (i.e., 13–15 kDa). So, it can be concluded that anticoagulant activity of *D. polylepis* was not mediated by PLA_2_s but by other toxins, perhaps SVMPs. Mascot searches with proteomics data retrieved from tryptic digests of nanofractionated venom toxin fractions collected in wells corresponding to the retention time of the bioactivity peak identified an SVMP toxin (species-specific venom gland transcriptome contig *D_polylepis*_T0167_SVMP), providing additional evidence that SVMP toxins may be at least partially responsible for the observed anticoagulant bioactivity (Figure 4; Table 1; Supplementary Figure S5).

**Figure 5:**
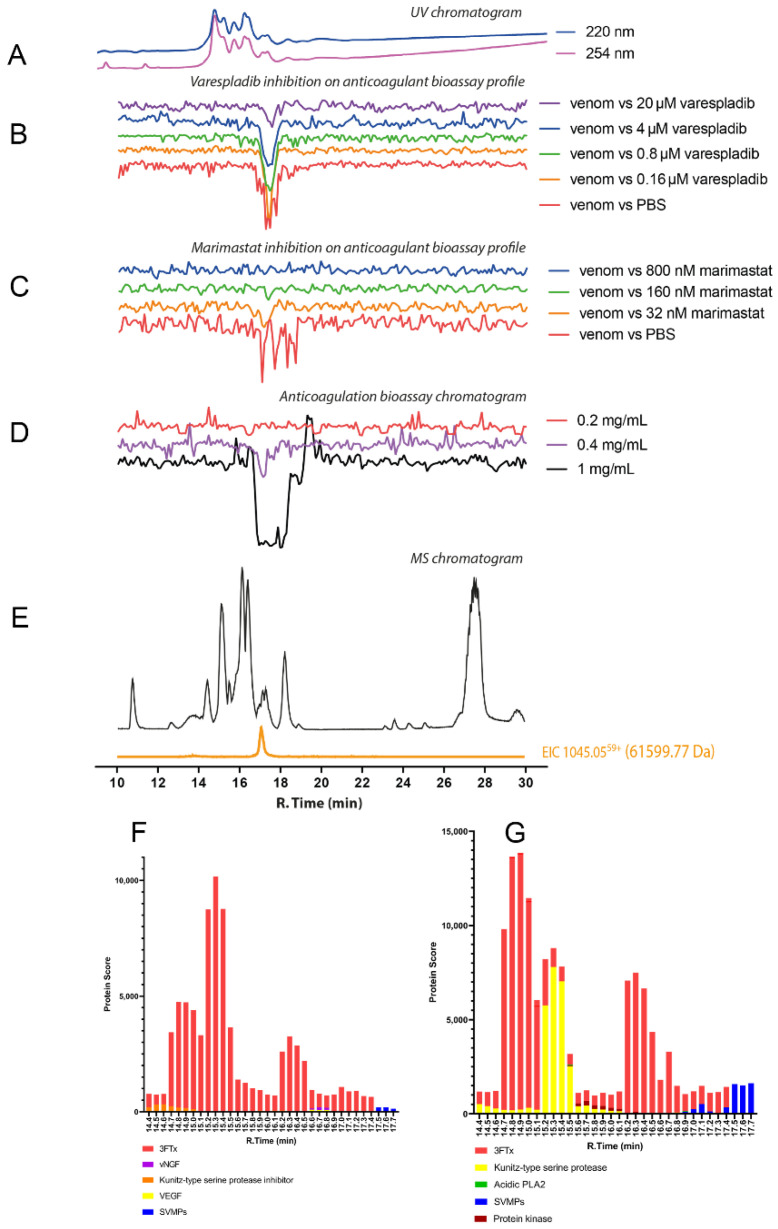
Correlation of LC-UV and MS chromatogram and bioassay chromatogram of *D. angusticeps* venom. (**A**) UV chromatogram of *D. angusticeps* venom (1 mg/mL, 50 μL injection volume, post-column split into 1:9 ratio. The smaller portion went to UV (220 and 254 nm recorded); and then to MS detection. Superimposed bioassay chromatograms resulting from analyses of *D. angusticeps* venom (1 mg/mL, 50 µL injection volume) in the presence of different concentrations of (**B**) varespladib and (**C**) marimastat. (**D**) Superimposed anticoagulation bioassay chromatograms resulting from analyses of several diluted *D. angusticeps* venom samples ranging from 1 mg/mL to 0.2 mg/mL (50 µL per injection). (**E**) Base peak chromatogram (BPC) and extracted ion currents (XIC) mass spectrometry. Bioassay chromatograms are correlated to UV and MS. Proteomic annotation of *D. angusticeps* venom using Mascot software against (**F**) the Swiss-Prot database and (**G**) species-specific venom gland transcriptomic-derived database. The protein score represents the probability that designated proteins, defined by elution time, are present in the sample.

**Figure 6:**
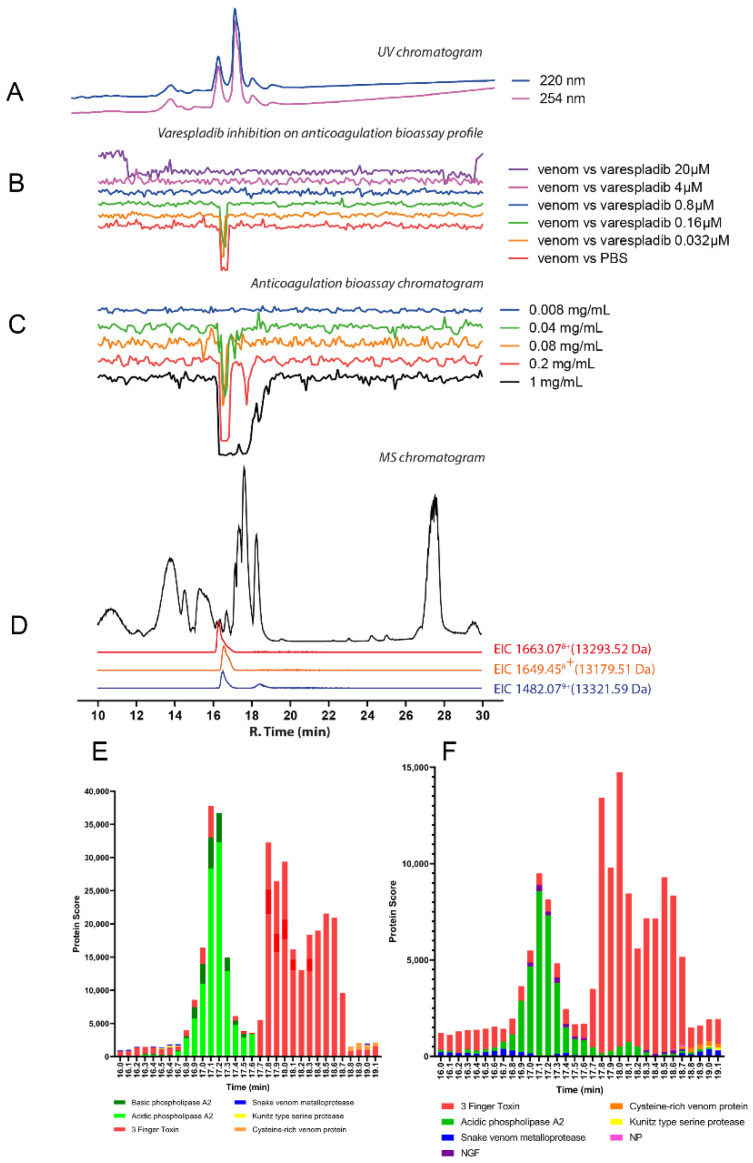
Correlation of LC-UV and MS chromatogram and bioassay chromatogram of *N. naja* venom. (**A**) UV chromatogram of *N. naja* venom (1 mg/mL, 50 μL injection volume, post-column split into 1:9 ratio of which the smaller portion went to UV (220 and 254 nm recorded); and then to MS detection. Superimposed bioassay chromatograms resulting from analyses of *N. naja* venom (0.2 mg/mL, 50 µL injection volume) in the presence of different concentrations of (**B**) varespladib. (**C**) Superimposed anticoagulation bioassay chromatograms resulting from analyses of several diluted *N. naja* venom samples ranging from 1 mg/mL to 0.008 mg/mL (50 µL per injection). (**D**) Base peak chromatogram (BPC) and extracted ion currents (XIC) mass spectrometry. Bioassay chromatograms are correlated to UV and MS. Proteomic annotation of *N. naja* venom using Mascot software against (**E**) the Swiss-Prot database and (**F**) species-specific venom gland transcriptomic-derived database.

Further, the resulting database hit shares 83.7% identity with the SVMP toxin “atragin” from *Naja atra* venom, which is known to exert anticoagulant activity [71]. Perhaps unsurprisingly, given the findings above, the SVMP inhibitor marimastat appeared to neutralise the detected negative anticoagulant peak at higher drug concentrations (Figure 5 and Figure 6; Supplementary Figures S12 and S13), although it is worth pointing out that we observed no evidence of crude venom neutralisation with this drug (Figure 3). However, the PLA_2_ inhibitor varespladib also abolished the anticoagulant toxin effects in the nanofractionation experiments, though the relatively low anticoagulation signals observed after fractionation in comparison to crude venom analysis make these results difficult to interpret, and thus observed inhibition could be the result of experimental conditions rather than direct toxin neutralisation.

#### 2.2.2. *Dendroaspis angusticeps*

The chromatographic bioassay profile of *D. angusticeps* showed a broad negative peak at the retention time region of 16.5–18.3 min at the highest (1 mg/mL; 50 μL per injection, 50 μg of venom proteins) concentration injected (Figure 5D; Supplementary Figure S6), correlating with our crude venom experiments that revealed more substantial anticoagulant venom activity in comparison with the congener *D. polylepis* (Figure 2). At reduced venom concentrations, only a sharper peak at around 16.5–17.3 min remained, with further dilution resulting in a significant loss of anticoagulant activity. At the retention time of 16.5–17.3 min, there were many coeluting venom toxins detected. The most noticeable traces observed by MS were from toxins with masses in the range of 6–7 kDa (which are probably 3FTxs and/or Kunitz-type serine protease inhibitors) and from a toxin with a mass around 60 kDa (possibly an SVMP) measured with relatively low intensity (Figure 5E; Table 1). A 4 μM concentration of varespladib seemingly inhibited the anticoagulant activity of the nanofractionated venom (at 1 mg/mL concentration), while as low as a 0.16 μM concentration of marimastat exerted a similar effect (Figure 5B,C). Marimastat thus inhibited the anticoagulant effect of *D. angusticeps* venom at lower doses, whereas the higher doses required for varespladib inhibition hint at non-specific effects. However, as with *D. polylepis*, neither drug substantially inhibited crude venom anticoagulant activity, even at high doses (Figure 3). From the resulting proteomics data, *D. angusticeps* venom toxins detected in the bioactivity region included two 3FTxs Toxin Tx7335 (3NOJ_DENAN; rt 17.1 min), and Rho-elapitoxin-Da1b (3SI1B_DENAN; rt 17.1 min) and the Kunitz-type serine protease inhibitor long epsilon-dendrotoxin His55 (VKTHE_DENAN; rt 16.5 min) (Figure 5F,G; Table 1; Supplementary Figure S13). Other minor components detected included a venom nerve growth factor (vNGF; rt 16.7 min) and SVMPs (rt 17.1 min, 61,599.77 Da, *D_angusticeps*_T0082), which could at least partially explain the inhibitory profile of marimastat in the nanofractionation experiments.

### 2.3. Nanofractionation, Identification, and Inhibition of Coagulopathic Cobra and Rinkhals Venom Toxins

#### 2.3.1. *Naja naja*

A broad negative peak defines the chromatographic bioassay profile of *N. naja* at a retention time region spanning 16.3–19.1 min (Figure 6C; Supplementary Figure S7). Venom dilution experiments showed a decrease of this broad negative peak into a single sharp peak with a retention time of 16.5 min, with anticoagulant activity still observed at a venom concentration of 0.04 mg/mL (50 μL per injection; 2 μg of venom proteins). The 0.2 mg/mL (50 μL per injection; 10 μg of venom proteins) venom concentration was selected for further use in inhibition experiments with varespladib. In contrast to the nanofractionation findings observed with the *Dendroaspis* spp., for all *Naja* species studied herein, marimastat failed to exert any inhibitory effect on anticoagulant venom activities at any of the tested concentrations (as was also the case for the crude venom analyses; see Figure 3), and thus will not be further discussed. Contrastingly, the anticoagulant activity of *N. naja* venom decreased markedly with increasing concentrations of varespladib, with complete neutralisation observed at a concentration of 0.8 µM (Figure 6B; Supplementary Figure S7). From the LC-MS data for *N. naja* (Figure 6D; Table 1), in the retention time region of 16.3–19.1 min, several proteins within the mass range of 6–7 kDa (i.e., 7803.47 Da; 7819.46 Da; 6750.33 Da; and 6733.32 Da) were detected, indicating the likely presence of 3FTxs. In addition, a protein with a mass of 13,321.59 Da, likely a PLA_2_, was also detected and eluted at the same retention time as the sharp bioactivity peak (~16.5 min). Proteomics data (Figure 6E,F; Table 1; Supplementary Figure S14) confirmed the presence of an acidic PLA_2_ (PA2A2_NAJNA, 13,321.59 Da) at the retention time of 16.5 min, which in combination with the varespladib inhibition data, strongly suggests that this PLA_2_ toxin is at least partially responsible for the observed anticoagulant venom effects.

#### 2.3.2. *Naja pallida*

**Figure 7:**
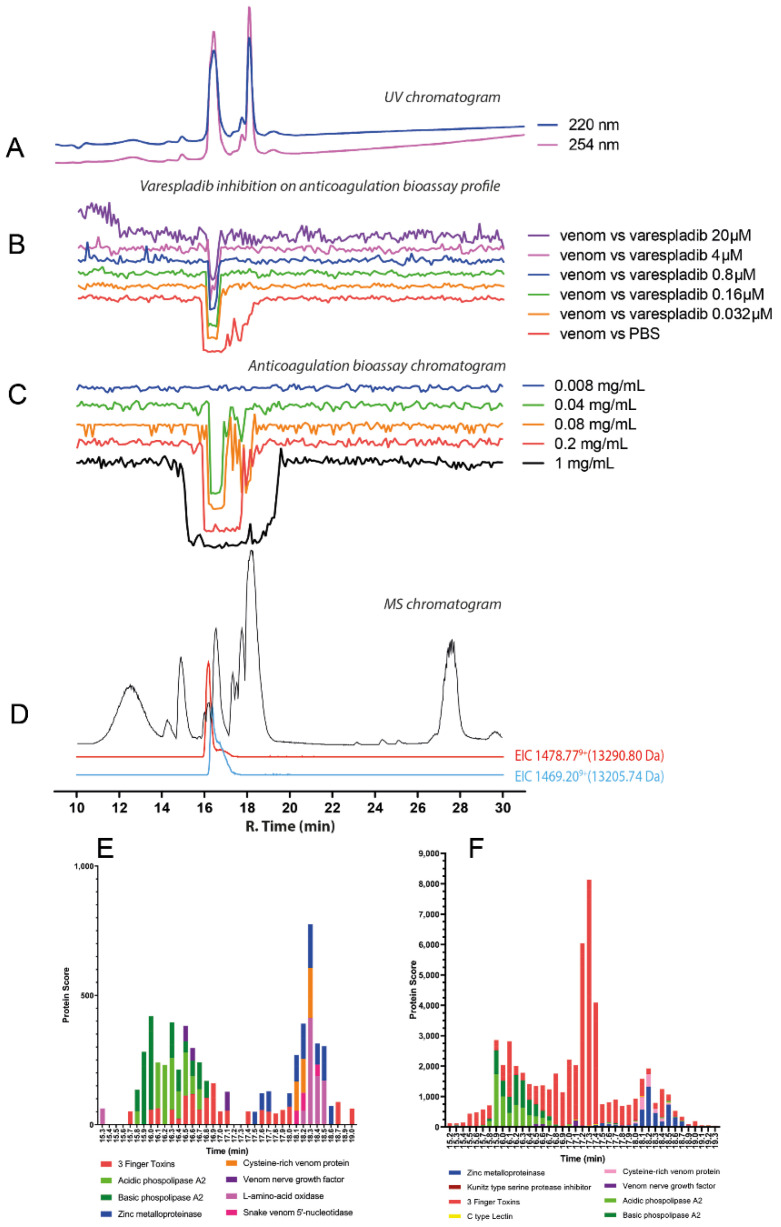
Correlation of LC-UV and MS chromatogram and bioassay chromatogram of *N. pallida* venom. (**A**) UV chromatogram of *N. pallida* venom (1 mg/mL, 50 μL injection volume, post-column split into 1:9 ratio of which the smaller portion went to UV (220 and 254 nm recorded); and then to MS detection. Superimposed bioassay chromatograms resulting from analyses of *N. pallida* venom (0.2 mg/mL, 50 µL injection volume) in the presence of different concentrations of (**B**) varespladib. (**C**) Superimposed anticoagulation bioassay chromatograms resulting from analyses of several diluted *N. pallida* venom samples ranging from 1 mg/mL to 0.008 mg/mL (50 µL per injection). (**D**) Base peak chromatogram (BPC) and extracted ion currents (XIC) mass spectrometry. Bioassay chromatograms are correlated to UV and MS. Proteomic annotation of *N. pallida* venom using Mascot software against (**E**) the Swiss-Prot database and (**F**) species-specific venom gland transcriptomic-derived database. The protein score represents the probability that designated proteins, defined by elution time, are present in the sample.

Similar to that of the congener *N. naja*, the chromatographic bioassay profile of *N. pallida* venom was defined by a broad negative peak at a retention time of 16.0–19.0 min (Figure 7C; Table 1; Supplementary Figure S8). Upon diluted venom analyses, the venom still retained anticoagulant properties down to a concentration of 0.04 mg/mL, though only a large peak at around 16.0 to 16.7 min remained. A 0.2 mg/mL concentration was chosen as the optimal concentration for subsequent assessment of anticoagulation inhibition by varespladib (Figure 7B; Supplementary Figure S8). From the LC-MS data (Figure 7D; Table 1), several toxins were found within the retention time region of 16.0–16.7 min and exhibited mass ranges between 6–7 kDa (6782.34 Da; 6815.32 Da), which indicated likely 3FTxs, and 13–14 kDa (i.e., 13,290.79 Da at rt 16.2 min and 13,205.74 Da and 13,320.73 Da at rt 16.4 min), indicating likely PLA_2_s. Although varespladib reduced the anticoagulant venom toxin activities upon increasing concentrations, with the broad peak observed in the venom only control significantly reduced by even the lowest concentration (0.032 μM) of varespladib tested, these experiments revealed a smaller anticoagulant peak that was not neutralised by varespladib even at the highest concentration tested (20 μM) (Figure 7B). From the proteomics data generated from the anticoagulant bioactivity region (Figure 7; Table 1; Supplementary Figure S15), both the basic phospholipase A_2_ (13290.79 Da, *N_pallida*_T0443) and basic phospholipase A_2_ nigexine (13,320.73 Da, PA2B_NAJPA) were detected.

#### 2.3.3. *Naja nigricollis*

**Figure 8:**
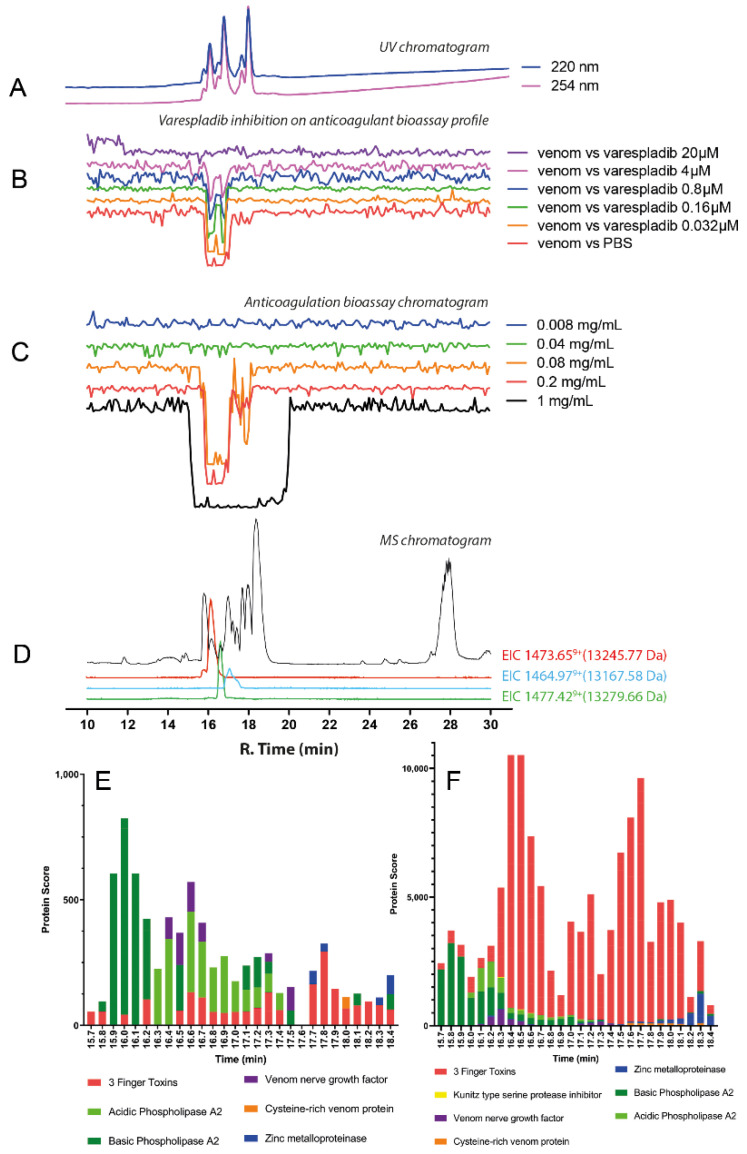
Correlation of LC-UV and MS chromatogram and bioassay chromatogram of *N. nigricollis* venom. (**A**) UV chromatogram of *N. nigricollis* venom (1 mg/mL, 50 μL injection volume, post-column split into 1:9 ratio and a smaller portion went to UV (220 and 254 nm recorded); and then to MS detection. Superimposed bioassay chromatograms resulting from analyses of *N. nigricollis* venom (0.2 mg/mL, 50 µL injection volume) in the presence of different concentrations of (**B**) varespladib. (**C**) Superimposed anticoagulation bioassay chromatograms resulting from analyses of several diluted *N. nigricollis* venom samples ranging from 1 mg/mL to 0.008 mg/mL (50 µL per injection). (**D**) Base peak chromatogram (BPC) and extracted ion currents (XIC) mass spectrometry. Bioassay chromatograms are correlated to UV and MS. Proteomic annotation of *N. nigricollis* venom using Mascot software against (**E**) the Swiss-Prot database and (**F**) species-specific venom gland transcriptomic-derived database. The protein score represents the probability that designated proteins, defined by elution time, are present in the sample.

The chromatographic bioassay profile of *N. nigricollis* (Tanzania) can be described as a broad negative peak at a retention time region of 16.2–18.1 min (Figure 8C). The venom still exhibited anticoagulant properties down to a venom concentration of 0.08 mg/mL. Nevertheless, at lowering venom concentrations, only two large sharp negative peaks at around 16.1 and 16.8 min are left in Figure 8C. These correspond clearly to the first two sharp peaks in the LC-UV chromatogram (Figure 8A). The LC-MS data (Figure 8D; Table 1) revealed a toxin found with a mass of 13,245.77 Da at a retention time of 16.1 min, while at 16.8 min, an exact mass of 13,279.66 Da was found. Varespladib was found to effectively neutralise the anticoagulant activity of *N. nigricollis* venom (Figure 8B; Table 1; Supplementary Figure S9). The broad anticoagulation peak changed into two individual anticoagulation bioactivity peaks which became smaller upon increasing varespladib concentrations. A 20 μM dose of varespladib effectively inhibited all the anticoagulation activities of the venom and the proteomics data (Figure 8E,F; Table 1; Supplementary Figure S16) detected the presence of a basic PLA_2_ (PA2B4_NAJNG) and an acidic PLA_2_ (Acidic Phospholipase A_2,_ *N_nigricollis*_T3199_T0053) at rt. 16.1 min and 16.8 min, respectively. These findings are identical to those we recently reported from the venom of Nigerian *Naja nigricollis* using the same methodology [38], where again a basic and acidic PLA_2_ with rts. of 16.1 and 16.8 min were responsible for the observed anticoagulant venom activity.

#### 2.3.4. *Naja haje*

**Figure 9:**
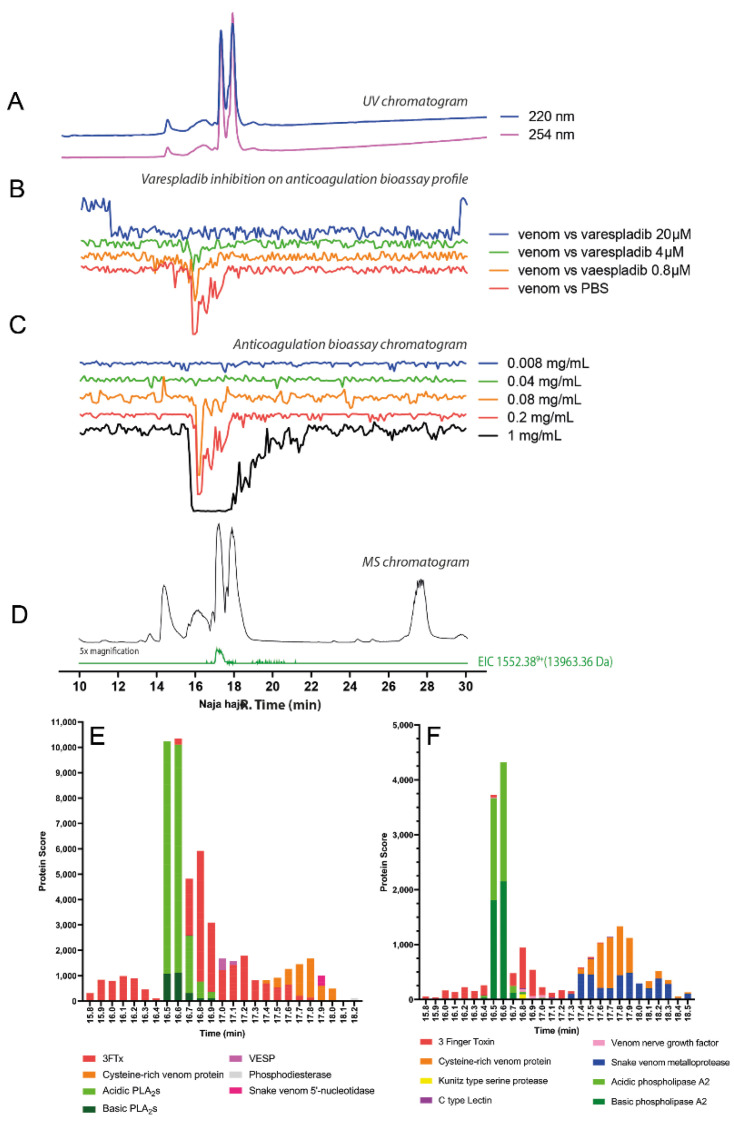
Correlation of LC-UV and MS chromatogram and bioassay chromatogram of *N. haje* venom. (**A**) UV chromatogram of *N. haje* venom (1 mg/mL, 50 μL injection volume, post-column split into 1:9 ratio of which the smaller portion went to UV (220 and 254 nm recorded); and then to MS detection. Superimposed bioassay chromatograms resulting from analyses of *N. haje* venom (0.2 mg/mL, 50 µL injection volume) in the presence of different concentrations of (**B**) varespladib. (**C**) Superimposed anticoagulation bioassay chromatograms resulting from analyses of several diluted *N. haje* venom samples ranging from 1 mg/mL to 0.008 mg/mL (50 µL per injection). (**D**) Base peak chromatogram (BPC) and extracted ion currents (XIC) mass spectrometry. Bioassay chromatograms are correlated to UV and MS. Proteomic annotation of *N. haje* venom using Mascot software against (**E**) the Swiss-Prot database and (**F**) species-specific venom gland transcriptomic-derived database. The protein score represents the probability that designated proteins, defined by elution time, are present in the sample.

The chromatographic bioassay profile of *N. haje* shows a broad negative peak at the retention time region of 15.5–18.5 min (Figure 9C). The very broad anticoagulant peak observed indicates the potential presence of closely eluting anticoagulant toxins. However, after performing experiments with diluted venom concentrations, a sharp bioactive peak was observed at retention time 16.3–16.7 min, and anticoagulant activity was detected down to venom concentrations of 0.08 mg/mL, thus indicating lower activity compared with congeners *N. pallida* and *N. naja*. Based on the LC-MS data, a protein detected with a mass of 13,963.38 Da at a retention time of 16.5 min, suggests a PLA_2_ toxin is likely associated with anticoagulant activity, though some 3FTxs were also detected around the same retention time (Figure 9D; Table 1). The superimposed bioassay chromatograms and LC-UV chromatograms from the varespladib inhibition experiments are shown in Figure 9B (Supplementary Figure S10). Figure 9B shows that the observed anticoagulant activity of *N. haje* venom decreased with an increasing concentration of varespladib. However, the anticoagulation activity of the venom was only fully neutralised by the highest concentration (20 μM) of varespladib, despite reduced venom activity in comparison with some other cobra venoms tested. While this data might hint at the presence of other toxins contributing to the anticoagulant effects observed, the processed proteomics data revealed a toxin eluting at a retention time of 16.5 min, which was annotated as an acidic PLA_2_ (PA2A3_NAJSG), suggesting once again that PLA_2_ toxins likely underpin anticoagulant venom activity in this genus (Figure 9E,F; Table 1; Supplementary Figure S17).

#### 2.3.5. *Hemachatus haemachatus*

**Figure 10:**
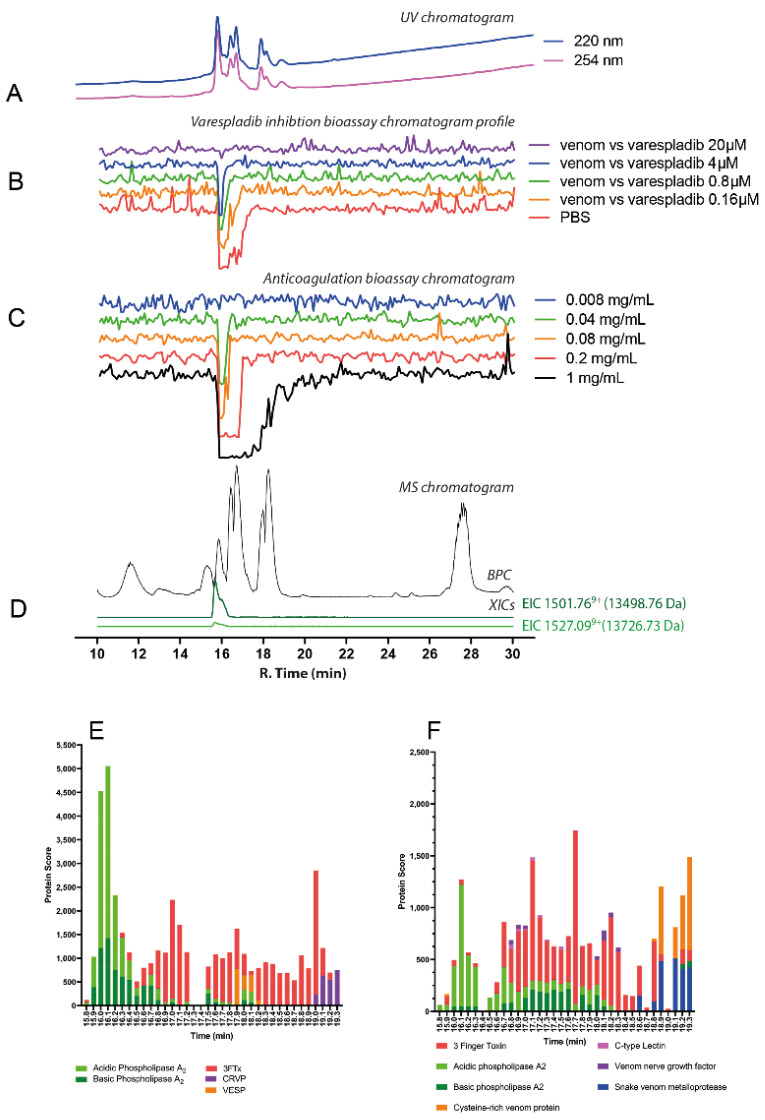
Correlation of LC-UV and MS chromatogram and bioassay chromatogram of *H. Haemachatus* venom. (**A**) UV chromatogram of *H. haemachatus* venom (1 mg/mL, 50 μL injection volume, post-column split into 1:9 ratio. The smaller portion went to UV (220 and 254 nm recorded), and then to MS detection. Superimposed bioassay chromatograms resulting from analyses of *H. haemachatus* venom (0.2 mg/mL, 50 µL injection volume) in the presence of different concentrations of (**B**) varespladib. (**C**) Superimposed anticoagulation bioassay chromatograms resulting from analyses of several diluted *H. haemachatus* venom samples ranging from 1 mg/mL to 0.008 mg/mL (50 µL per injection). (**D**) Base peak chromatogram (BPC) and extracted ion currents (XIC) mass spectrometry. Bioassay chromatograms are correlated to UV and MS. Proteomic annotation of *H. haemachatus* venom using Mascot software against (**E**) the Swiss-Prot database and (**F**) species-specific venom gland transcriptomic-derived database. The protein score represents the probability that designated proteins, defined by elution time, are present in the sample. Note: after nanofractionation analytics, no procoagulant toxin peaks were observed (not shown in the figure). SVSPs and other mainly larger toxins can denature during the chromatographic separation thereby losing their biological activity (which we have observed in other ongoing research projects).

The chromatographic bioassay profile of *H. haemachatus* was defined by a broad negative peak at a retention time region of 15.8–17.9 min, similar to that of related cobra species (*Naja* spp.) (Figure 10C). Venom dilution analyses showed anticoagulant activity was retained down to concentrations of 0.08 mg/mL, where a sharp, rather than broad, negative peak with a retention time of 16.0 min was visible. In line with data from *Naja* spp., the resulting mass data (Figure 10D; Table 1) detected several proteins within the mass range of 6–7 kDa (6788.43 Da, rt. 16.4 min; 6832.45 Da, rt. 16.7 min; and 6779.36 Da, rt. 17.8) which are most probably 3FTxs. A protein with a mass of 13,498.76 Da was also detected, and likely to be a PLA_2_. This protein was eluted with essentially the same retention time as the bioactivity peak at 15.8 min. Moreover, varespladib dose-dependently inhibited the anticoagulation activity observed in *Hemachatus haemachatus* venom, though only the high 20 μM concentration was capable of completely inhibiting the anticoagulation activity (Figure 10B; Supplementary Figure S11). From the proteomics data (Figure 10E,F; Table 1; Supplementary Figure S18), *Hemachatus haemachatus* venom mainly consisted of 3FTxs which eluted within the retention time profile of 16.4–18.2 min, though a PLA_2_ toxin was identified (15.8 min, PA2B1_HEMHA, basic phospholipase A_2_) at the exact retention time of the anticoagulation bioactivity peak.

## 3. Discussion

With the exception of the two mamba species studied, the elapid venoms in this study showed strong anticoagulant activity [20], particularly in the venoms of spitting cobras [25,72], but has, until now, been unverified in mambas. Both the crude coagulation assays with inhibitors and the nanofractionation results described here implicate PLA_2_s as the main anticoagulant agent in cobra venoms. This is unsurprising; cobra venom PLA_2_s have been shown to impede the process of coagulation through the inhibition of platelet aggregation [30], by non-enzymatic binding to the clotting factor Xa [25], the disruption of the tenase and prothrombinase complexes [33,34] and the lysis of fibrin.

Contrasting with the cobras, the identity of anticoagulant venom toxins in mamba venoms remains uncertain. Although inhibition of anticoagulation by varespladib was shown for nanofractionated mamba venoms at higher drug concentrations, no inhibitory effects were observed on crude mamba venoms in the plasma bioassay. The nanofractionation results are challenging to interpret due to the reduced anticoagulation levels observed compared to crude venom analyses. It may be that anticoagulant venom toxins in mamba venoms are prone to denaturation during the reversed-phase separation process. The inhibition of the remaining lower activity observed at higher drug concentrations may be a non-specific effect, thereby rendering these results inconclusive.

Further, proteomic data has previously demonstrated that mamba venoms lack any abundant PLA_2_s [15], which are known to be stable during reversed-phase LC separations in the proteomes of these species. Thus, other toxin families are highly likely to be responsible for the anticoagulation effects observed in mamba venoms. Our attempts to identify the toxin(s) causing the small anticoagulant peak observed in the *D. polylepis* bioassay chromatograms (at a retention time of 16.5 min) resulted in the detection of a bioactive venom metalloproteinase (SVMP) but the effect can also be caused by another protein that coeluted at the same retention time. Although strong matches to any elapid toxin present in the UniProt database were lacking, when searched against a *D. polylepis* venom gland transcriptome database [15], besides several 3FTXs eluting within the bioactivity range, one SVMP was identified from proteomic analysis, and this had a retention time consistent with the mass range of SVMPs. Despite this, the lack of inhibition of venom activity from all doses of marimastat suggests that SVMPs do not play a significant role in the anticoagulant activity in mamba venoms unless these SVMPs are unaffected by this broad-spectrum metalloproteinase-inhibiting drug. The potential roles of 3FTxs and Kunitz-type serine protease inhibitors in the observed anticoagulant activity of mamba venoms remain to be seen, and it may be worth isolating these toxins to purity followed by performing coagulation and other bioassays in the future. However, it is also possible that these toxins are coincidentally co-eluting with the actual bioactive toxin(s) in the experiments described herein.

Our findings show that the small molecule inhibitor varespladib can neutralise the potent anticoagulation effects often observed from cobra venoms. However, for mamba venoms, neither varespladib nor marimastat provided any inhibitory effects against venom-induced anticoagulation. These findings suggest that distinct toxin types are likely responsible for the effects observed in the mambas, and that anticoagulant venom effects likely evolved independently on multiple occasions in African elapids. Previous work investigated the effectiveness of antivenom on neutralising modulation of coagulation effects by snake venom using a similar analytical approach [53,63,73]. This study investigates how the small molecule inhibitors varespladib and marimastat can neutralise the same coagulant modulating effects caused by toxins in cobra and mamba venoms. Although not elaborately discussed, this research includes for all venom under study a comprehensive set of venom proteomics data. The summarised venomics data are given in this paper in the figures, while the complete detailed proteomics datasets are presented in the supporting information.

Marimastat failed to reduce the anticoagulant activity of the cobra venoms used in this study, with the exception of *Naja haje*, in which the highest dose (100 µM) elicited a reduction in anticoagulant activity comparable with that of varespladib at doses of as little as 0.8 µM (see Supplementary Figure S2). This conflicts with previous work performed on spitting cobras which showed a significant reduction in anticoagulant activity upon the application of marimastat and the related matrix metalloproteinase inhibitor prinomastat in most species tested [39]. The lack of inhibitory effect of marimastat observed in most species tested here could reflect a denaturation process during nanofractionation, which SVMP toxins would be subjected to, although this does not explain the lack of inhibition observed in our crude venom experiments. Thus, we conclude that, although marimastat exhibits considerable promise as a lead repurposed drug for snakebite envenoming caused by SVMP-rich viper venoms [37,74], its potential utility against elapid-induced coagulopathy appears limited, though it may be effective in cases of snakebite by *N. haje* in higher doses. The proportion of *N. haje* venom that is made up of SVMPs is debatable, ranging from 0 to 10% of the toxic component [14,75]; however, PLA_2_s also make up a considerably small amount of the venom; 0.1–4% [14,75], which begs the question of why varespladib is still effective at neutralising this venom at all and more so than marimastat. Marimastat has been shown to work at neutralising SVMP-mediated toxicity and lethality, especially when used in conjunction with antivenom [53,65,66,67,68,69], however this study finds no clear justification for its use as a treatment for incidents of elapid envenomation, likely due to lack of medically-important SVMPs in elapid venoms compared to those of vipers. Thus, marimastat was not included in the part of the study dealing with separated and fractionated toxins as it had not much effects on the anticoagulation evoked by the elapid venoms under study when measured as crude venoms.

Varespladib has shown considerable potential as a treatment for snakebite envenoming in previous laboratory studies, including abrogating the enzymatic effects of PLA_2_s from several vipers and the elapid *Oxyuranus scutellatus,* and Asian cobras [25,37,58,63], and can neutralise the haemotoxic effects from many elapid species [25,38,39,63]. However, the drug concentrations required to completely inhibit the anticoagulant activity of PLA_2_ toxins in vitro were found to vary extensively; between 0.8 μM to 20 μM in some studies [63,64]. In this study, varespladib demonstrated potent inhibition of in vitro anticoagulation activities for crude cobra venoms at doses of 4 μM and above, and for the nanofractionated toxins from these venoms. For all cobras under investigation, one to a few PLA_2_s could be identified as the likely bioactive toxins causing the anticoagulation activities observed, and which could all be effectively inhibited by varespladib. Varespladib is a drug candidate that has undergone phase I and II trials for the treatment of cardiovascular diseases [76,77,78] and, more recently, has acquired significant interest as a candidate drug for the treatment of snakebite, together with its orally available derivative, methyl-varespladib. Following extensive in vitro and preclinical in vivo successes, methyl-varespladib has recently entered into phase II clinical trials for snakebite indication in the USA (https://clinicaltrials.gov/ct2/show/NCT04996264, accessed on 8 October 2021). Thus, these PLA_2_ inhibitors are currently lead candidates for the treatment of PLA_2_-mediated toxicities (Clare et al., 2021), and here we provide additional evidence of their potential utility against the anticoagulant activities of the cobra, though not mamba, venoms. This supports a growing amount of evidence that varespladib is a good candidate as a snakebite therapeutic drug, alongside conventional antivenom treatment, that may aid in improving the outcomes for victims of elapid envenomation.

The question arises of why typically neurotoxic elapids have anticoagulant components in their venoms. Perhaps the anticoagulant effects of said toxins aid in spreading other neurotoxic components, such as 3FTXs, to encourage systemic envenomation. This may be evidenced by studies that show that lethal doses of venoms from the elapids *Micrurus fulvius* and *Oxyuranus scutellatus*, and neurotoxically-acting viper *Vipera berus nikolskii* can be neutralised by the addition of varespladib to inhibit the action of PLA_2_s [57,58,59,79,80]. Further, varespladib can delay the onset of lethality caused by metalloproteinase-rich venoms in vivo, again suggesting a role for PLA_2_ inhibition in mitigating toxin spread or dampening the severity of pathology [53]. The difficulty with this hypothesis is that PLA_2_s are highly flexible in function, being able to act directly to cause presynaptic neurotoxicity or inhibit coagulation or contribute to cytotoxic effects [81]. Indeed, it may be that this multifunctionality of PLA_2_s, which can act directly and indirectly, could be a contributing factor to the potent neurotoxicity of certain elapid snakes. However, what is clear is that African elapids have independently evolved distinct anticoagulant toxins. While these are unquestionably PLA_2_s in cobras (and which are particularly abundant and bioactive in spitting cobras) [20,38,72], the identity of those in mambas is much less clear, though based on findings herein, they seem likely to be toxins distinct from PLA_2_s [15]. The multiple independent origins of anticoagulants in these elapid snakes hint at a biologically relevant role in this activity for prey capture. Future clinical research will hopefully also contextualise the relevance of such findings in human snakebite envenomings.

## 4. Conclusions

This study investigated the anticoagulant activities of mamba and cobra venoms. These effects could be reduced dose-dependently with the addition of varespladib in the case of cobras and rinkhals. For all cobras, strong anticoagulation effects were observed. In case of the mambas, of the two mambas under study, *Dendroaspis polylepis* and *Dendroaspis angusticeps*, weaker anticoagulation effects were observed, of which the *Dendroaspis angusticeps* venom was the more potent. Neither varespladib nor marimastat neutralised the coagulation modulation evoked by mamba venom fractions tested in this study. These observations, combined with nanofractionation data identifying fractions likely to be involved in such activity, implicates the role of PLA_2_s from cobra, but not mamba, venoms in inhibiting blood coagulation pathways. The toxins responsible for anticoagulant effects caused by mamba venoms remain unclear. However, collectively these findings suggest that these two groups of medically important elapid snakes have independently evolved anticoagulant venom activities. Although not elaborately discussed, this research includes for all venoms under study a comprehensive set of venom proteomics data. The summarised venomics data are given in this paper in the figures, while the selected retention time detailing proteomics datasets are presented in the supporting information.

## 5. Materials and Methods

### 5.1. Chemical and Biological Reagents

Water was purified with a Milli-Q Plus system (Millipore, Amsterdam, The Netherlands). Dimethyl sulfoxide (DMSO) was supplied by Riedel-de-Haen (Zwijndrecht, The Netherlands). Acetonitrile (ACN) LC/MS grade was obtained from Concord, NC, USA. Formic acid (FA, MS grade) and acetic acid (AA) were purchased from Biosolve (Valkenswaard, The Netherlands), while CaCl_2_, K_2_HPO_4_, KH_2_PO_4_, NH_4_HCO_3_, phosphate buffer saline (PBS) tablets, and other salts used for buffer preparation were analytical grade and purchased from standard suppliers (Merck, Kenilworth, UK; Fluka, Bucharest, Romania; or Sigma-Aldrich, Darmstadt, Germany). The PLA_2_ inhibitor varespladib, the SVMP inhibitor marimastat, alkylating agent iodoacetamide, and argatroban control were purchased from Sigma-Aldrich, Darmstadt, Germany. A 0.1 mg/mL stock solution of argatroban was used for aligning the bioassay chromatogram with UV traces according to previously-defined methods [38]. The 1000 µM stock solution of varespladib and marimastat were made in DMSO and kept at −20 °C for short term storage and at −80 °C for longer-term storage. Bovine plasma was sourced from Biowest (Nuaille, France). The plasma was defrosted in a water bath at 37 °C, centrifuged, and aliquoted (approximately 10 mL aliquots in 15 mL falcon tubes) and kept at −20 °C until use. Lyophilised trypsin Gold (mass spectrometry grade) enzyme was purchased from Promega Corporation, Madison, USA. The trypsin was reconstituted in 50 mM acetic acid to obtain 1 µg/µL concentration and was aliquoted (10 µL in 1 mL Eppendorf tubes). The aliquoted solution was kept at −80 °C.

Lyophilised venom from black mamba (*Dendroaspis polylepis,* Tanzania), Eastern green mamba (*Dendroaspis angusticeps,* Tanzania), Indian cobra (*Naja naja,* captive-bred), red spitting cobra (*Naja pallida,* Tanzania), black-necked spitting cobra (*Naja nigricollis,* Tanzania), Egyptian cobra (*Naja haje*, Uganda) and rinkhals (*Haemachatus haemachatus,* South Africa) were sourced from snakes held in the Herpetarium of the Centre for Snakebite Research and Interventions at the Liverpool School of Tropical Medicine, UK. Venoms were stored at 4 °C until reconstituted, whereafter storage was at −80 °C.

### 5.2. Crude venom Coagulation Bioassaying and Inhibition with Varespladib and Marimastat

Plasma clotting assays were performed on citrated bovine plasma, following protocols previously outlined [82]. Plasma was stored at −80 °C and defrosted in a water bath at 37 °C, before being centrifuged at 2000 rpm for 4 min to remove the precipitate. The supernatant was then pipetted into a 50 mL falcon tube and kept on ice until use. For crude venom assays, venom samples were diluted with PBS, resulting in the following final assay doses: 1, 10, 50, and 100 ng. Samples were pipetted in 10 µL volumes, in replicates of 7, onto a Greiner Bio-One transparent 384-well microplate. Negative controls contained 10 µL PBS (i.e., no venom). To all wells, 20 µL of 20 mM calcium chloride was added using a Thermo Scientific Multidrop™ 384 Labsystems multidrop pipette. The plate was spun down for 5–10 s on a Benchmark Scientific PlateFuge™ MicroPlate MicroCentrifuge to remove bubbles. The multidrop was then flushed with deionised water before using it to add 20 µL of plasma into each well. Plates were read kinetically at a wavelength of 595 nm and a temperature of 25 °C using a FLUOstar Omega plate reader, with a run time set to 40 cycles at the minimum possible cycle time for the plate occupancy (141 s). Clotting activity was measured as the mean absorbance curve (area under the curve (AUC)) of each venom concentration in the relevant clotting period (determined by the clotting curve of the control to be between 24 and 71 min), standardised as a percentage of the mean AUC of the negative control in the same period. The crude clotting curves determined that 100 ng of venom in each plate well should be for the small molecule inhibition assays to follow.

To test the efficacy of small molecule inhibitors at preventing the anticoagulant activity of elapid venoms, dilutions of the SVMP inhibitor marimastat or the PLA_2_ inhibitor varespladib were made up using DMSO, to make final assay reaction concentrations of 0.8 µM, 4 µM, 20 µM, and 100 µM. Inhibitor-containing samples consisted of 6.25 µL venom (concentration 10 µg/mL) and 3.75 µL inhibitor. Positive controls consisted of 100 ng of crude venom and negative controls of 10 µL PBS only. All samples were pipetted into a 384-well plate in quadruplicate and the plate was incubated at 37 °C for 30 min to allow the inhibitors to take effect. As previously described, 20 µL of 20 mM calcium chloride was then added to the plate via the multidrop, centrifuged in a microcentrifuge, and then 20 µL of thawed bovine plasma was added. The plate was read in the FLUOstar at 595 nm for 45 cycles at the minimum possible cycle time for the plate occupancy (147 s). Clotting activity was measured as the mean area under the curve (AUC) of each sample type in the relevant clotting period (determined by the clotting curve of the control to be between 22 and 71 min), standardised by the mean AUC of the negative control in the same period. Readings were captured in at least triplicate. One-way analysis of variance (ANOVA) analysis was performed on clotting activity in GraphPad Prism 5.0, with Dunnett’s Multiple Comparison post-hoc tests used to determine the significance of the reduction of anticoagulant activity between the control well and the inhibitor for each species. These data were used to generate dose–response data for each drug and venom.

### 5.3. Coagulation of Nanofractionated Elapid Venom

#### 5.3.1. An Overview of the Workflow

The workflow of the analysis of nanofractionated venom toxins on coagulation activity, the parallel acquired LC-MS-based accurate mass determination of the separated toxins, and the inhibition of their anticoagulation activity by varespladib and/or marimastat is represented in the lower part (ii) of Figure 1. Nanofractionation of venom (50 µL injections) was performed into 384-well plates for subsequent plasma coagulation assaying of all high-resolution collected fractions. This bioassay measures differences in coagulation velocity between control wells, which produce a typical coagulation profile, and wells containing bioactive venom toxins [38,82]. Plotting end-point measurement absorbances measured in each well against the time of fractionation results in negative peaks for anticoagulant toxins in these wells, where clotting is thus reduced or even fully inhibited. By plotting the slopes of the coagulation curves kinetically measured for each well in time against fractionation time, for procoagulant venom toxins, positive peaks are observed in these so-called bioactivity chromatograms. The parallel obtained LC-UV, and LC-MS data are plotted as superimposed chromatograms for matching UV peaks and exact masses (using the Total Ion Current, TIC, and Extracted Ion Currents, XICs) with negative bioactivity peaks observed. For analysis of the small molecule inhibitors varespladib and marimastat on their capability of neutralising elapid venom toxin-induced coagulation activities, as observed in the chromatographic coagulation bioassay profiles, each venom was nanofractionated at its optimal concentration (i.e., that concentration resulting in good bioactivity signals, but not resulting in peaks that are too broad). Nanofractionated venom toxins in the wells of the (vacuum centrifuge freeze-dried) well plates were then pre-incubated using different concentrations of varespladib (for cobras and mambas) or marimastat (for mambas), before the coagulation activity assay was performed. The resulting bioassay chromatograms of the serially diluted varespladib and marimastat experiments per venom were analysed and presented as superimposed chromatograms.

#### 5.3.2. Instrumental Setup for HPLC/MS-Nanofractionation

LC-MS carried out the separation of venom toxins for the post-column assay with high-resolution nanofractionation. The samples (50 µL) were injected into a Shimadzu SIL-20A autosampler, and LC separation was performed with an LC system controlled via Shimadzu Lab Solution software. the gradient was set using a binary Shimadzu LC-20AB pump (pump A and B) at a total flow rate of 0.5 mL/min. Mobile phase A was water-ACN-FA (98:2:0.1, *v*/*v*/*v*) and mobile phase B was water-ACN-FA (2:98:0.1, *v*/*v*/*v*). The following gradient was used: 0% to 10% B (10 min), 10% to 95% B (20 min), 95% B (2 min), 90% to 0% B (7 min), 0% B (2 min). A 100 × 4.6 mm ID analytical column with XbridgeTM BEH300 reversed-phase C18 material (particle diameter 3.5 µm and pore size 300 Å) was used for separation. The column eluate was split in a 1:9 ratio using a low-dead-volume flow splitter. The smaller flow (0.05 mL/min) was led to a Shimadzu SPD 20A UV-Vis detector and Bruker Maxis mass spectrometer (MS). For mass analysis, the Maxis HD mass spectrometer was equipped with an electrospray ionisation source (ESI) and operated in positive ion mode. The parameters of the ESI source were source temperature 200 °C; capillary voltage 4500 V; dry gas flow 4.0 L/min; mass range 500–3000 *m*/*z* with a data-sampling time of 1 s. Protein mass (Da) were calculated using Data Analysis 5.0 (Bruker, Darmstadt, Germany). The larger eluate flow was nanofractionated (6 s/well) onto transparent 384-well plates (Greiner Bio-One, Alpen aan den Rijn, The Netherlands) controlled by Ariadne software. After nanofractionation, the plates were overnight vacuum centrifuged to dryness at room temperature using a Christ Rotational Vacuum Concentrator RVC 2–23 CD Plus (Salm en Kipp, Breukelen, The Netherlands), with a cooling trap at −80 °C. The plates were then stored at −80 °C until use.

#### 5.3.3. Coagulation Activities of Nanofractionated Venom an Inhibition by Varespladib and Marimastat

All measurements of coagulation activity were carried out on vacuum centrifuged freeze-dried 384-well plates and performed in duplicate. The plates were first placed at room temperature for 30 min toe defrost, and assay preparation was performed at room temperature. For measurements, a working solution of 20 mM CaCl_2_ was prepared (2 weeks expiration time when kept at 4 °C) and frozen aliquots of citrated bovine plasma were rapidly defrosted in a water bath at 37 °C until thoroughly defrosted. After defrosting, the plasma was centrifuged (2000 RPM for 4 min) briefly to remove any particulate matter. The mixing of plasma with calcium chloride solution was carried out by first transferring 20 μL of 20 mM calcium chloride solution to the wells of the well plate followed by adding 20 µL citrated bovine plasma, using a Thermo Scientific Multidrop™ pipetting robot. The preparation and mixing process was carried out within 5 min.

To neutralise potential PLA_2_s and/or SVMP activities, the respective inhibitors varespladib or marimastat were added to the well plate wells prior to adding calcium chloride and plasma. For this, serial dilutions (0.16 µM; 0.8 µM; 4 µM; 20 µM; and 100 µM) of varespladib and marimastat prepared in PBS were used. With these solutions, 10 µL of inhibitor solution was added to rows 8–18 of well plates with nanofractionated venom. Additionally, PBS was used as a negative control in rows 4–7 and in rows 19–22. Plates were then spun down by centrifuging at 2000 RPM for 2 min and then incubating at room temperature for 30 min with gentle shaking (using a shaker at 60 RPM). The resulting final assay concentrations of varespladib and marimastat were 0.032 µM; 0.16 µM; 0.8 µM; 4 µM; and 20 µM. Absorbance was measured at a wavelength of 595 nm by a Thermo Scientific Varioskan Lux™ Platereader using SkanIt 4.1 software. Measurement was performed at room temperature in one kinetic loop consisting of 80 readings within 1.5 h. Two data-processing methods were carried out to produce: (1) single reading at 80th reading for anticoagulation (i.e., endpoint read), (2) slope of a reading range for procoagulation, in case any procoagulation was observed. No significant procoagulation was observed in any of the experiments conducted in this study.

### 5.4. Proteomics Analysis

#### 5.4.1. In-Solution Tryptic Digestions from 384-Well Plates Containing Nanofractionated Venom Toxins

Vacuum centrifuged well-plates containing nanofractionated venom toxins were removed from the freezer and defrosted at room temperature in a closed flow cabinet. Wells containing bioactive of interest were reconstituted in 40 μL milli-Q water. Plates were then centrifuged gently at 1000 RPM for 30 sec using a well plate centrifuge (Eppendorf^®^ Centrifuge 5810R, Nijmegen, The Netherlands), followed by careful shaking at 60 RPM for 30 min on a plate shaker (IKA^®^ KS 4000 IC Control, Staufen, Germany). Digestion buffer was prepared by mixing a reducing agent (0.5% β-mercaptoethanol in milli-Q water) and freshly prepared AmmBi buffer pH 8.2 (25 mM ammonium bicarbonate) at a 1:9 ratio. Thereafter, 50 μL of digestion buffer and 30 μL of reconstituted venom proteins were transferred to an Eppendorf tube, followed by vortexing at 1000 RPM for 10 sec using a tube centrifuge (Prism^®^ mini labnet). The samples were then reduced at 95 °C for 10 min using a dry block heating thermostat (Biosan^®^ Bio TDB-100) for incubation. Next, 9 μL of alkylation agent (100 mM Iodoacetamide) was added followed by incubation for 30 min at room temperature in a closed cabinet to prevent light degradation of the alkylating agent. Following incubation, trypsin stock solution (1 μg/μL in 50 mM acetic acid solution) was diluted by AmmBi buffer pH 8.2 to obtain a 0.1 μg/μL concentration. Then, 5 μL of trypsin solution was added to each Eppendorf tube, vortexed, and subsequently incubated at 37 °C for 1.5–3.0 h using an incubator (Memmert, Schwabach, Germany). After that, an additional 5 μL of trypsin solution was added to the Eppendorf tubes and the samples were incubated at 37 °C overnight inside the incubator. After this incubation step, 10 μL of 5% formic acid solution was added to each tube to quench the digestion process. The resulting samples were finally transferred to autosampler vials and analysed by nanoLC–ESI-MS/MS.

#### 5.4.2. Instrument Setup for Proteomics

An Ultimate 3000 nano HPLC module (Thermo Scientific, Waltham, MA, USA) coupled to a Bruker TIMS-TOF Mass Spectrometer (Bruker Daltonics, Bremen, Germany) was used. Sample (1 µL) was injected with a WPS-3000(RS) autosampler, and nanoLC separation was performed with a nanoLC system controlled via Chromeleon 7.2 SR4 MUb software. The gradient was set using a nanoLC binary pump (A and B) at a total flow rate of 0.5 µL/min. Mobile phase A was water-FA (100:0.1, *v*/*v*), and mobile phase B was water-ACN-FA (20:80:0.05, *v*/*v*/*v*). The system was also equipped with a loading pump, for which solvent water-ACN-FA (99:1:0.05, *v*/*v*/*v*) was used. The following gradient was used: 1% B (10 min), 1–20% B (5 min), 20–50% B (30 min), 50–85% B (1 min), 85% B (5 min), 85–1% B (0.5 min), and 1% B (9.5 min). For sample trapping, an Acclaim PepMap 100 reversed-phase C18 trapping column (particle diameter 5 µm; pore size 100 Å; and column dimensions of 5 × 0.3 mm) was used. An Acclaim PepMap 100 reversed-phase C18 analytical column (particle diameter 2 µm; pore size 100 Å; and column dimensions of 150 × 0.75 mm), was used to separate the peptides in the samples subsequently. Both the analytical column and the trapping column were placed in a column oven, of which the temperature was set at 45 °C. The column eluate was transferred to a Bruker TIMS-TOF Mass Spectrometer (Bruker Daltonics, Bremen, Germany) equipped with a captive spray ionisation source in positive ion mode. The parameters of the CSI source were source temperature, 150 °C; desolvation temperature, 180 °C; capillary voltage, 1300 V; gas flow, 3 L/min. The monitored mass range was *m/z* 300–3000 with a data-sampling time of 0.5 s. The collision energy was 10 eV with prepulse storage of 10 µs. For MS/MS, the following parameters were used: precursor ion list 300–3000 *m/z*; number of precursor ions 3; threshold 250; preferred charge states +1 up to +3; fixed MS/MS acquisition 2 Hz (0.5 s).

#### 5.4.3. Proteomics Data Interpretation and Analysis

The tryptic digests were analysed with nanoLC–MS/MS, and the obtained MS/MS data for each of the samples were converted into mascot generic format files (MGF) by using Bruker DataAnalysis software. The obtained MGF files were subjected to Mascot database searching by using two different databases: the Swiss-Prot database and a species-specific database (MassIVE accession numbers MSV000081885 and MSV000080491, NCBI sequence read archive numbers SRX5026268, SRX7700833, SRX7700829, SRX5026269, SRX2768426, and SRX2768427). A detailed method for performing the in-solution tryptic digests is described in Section 5.4.1. The following parameters were used for the Mascot searches: (1) since iodoacetamide was used as an alkylating agent, fixed modification: carbamidomethyl (C) was chosen (adding 34 Da to methionine residues); (2) variable modifications: amidation (C-term) and methionine oxidation (M), (3) peptide tolerance of ±0.1% and MS/MS tolerance of ±0.05 Da, (4) peptide charges of +1, +2, and +3 allowed.

## Figures and Tables

**Table 1:** Overview of tentatively assigned venom toxins associated with the observed anticoagulation peaks. The table shows *m*/*z* values, including the charge state measured for each *m*/*z* value of intact toxins observed in MS, their retention times, and the calculated accurate mass for each toxin. Moreover, proteomics-derived Mascot (Swiss-Prot) protein identification and Mascot-derived corresponding masses are shown, as a match to the species-specific venom gland transcriptome databases. Hits were obtained by database searches of nanoLC–MS/MS data measured from tryptic digests of the respective venom toxins after nanofractionation and collecting these venom toxins from their respective wells in which they were fractionated. As many toxins, especially the larger toxins such as SVMPs, can have post-translational modifications (PTMs) such as glycosylation, the molecular mass (mol. mass (in Da)) found by high resolution mass spectrometry does not always match the Mascot exact mass (in Da).

Species	R. Time (min)	*m*/*z*	Charge	Mol. Mass (Da)	Protein Identification/Mascot Hits	Mascot Exact Mass (Da)	Species-specific Database Hits	Toxin Class
*Dendroaspis polylepis*	14.4	1019.93	7+	7129.47	Three-finger toxin	N.A.	N.A.	3FTx
	15.3	1007.31	7+	7040.16	Three-finger toxin	N.A.	N.A	3FTx
	15.8	1006.03	7+	7031.17	3SLS_DENPO (Calciseptin) *Dendroaspis polylepis*	7031.21	D_polylepis_T0010	3FTx
	16.4	999.34	8+	7982.68	3L24_DENPO (Alpha-elapitoxin-Dpp2d) *Dendroaspis polylepis*	7986.68	N.A.	3FTx
	16.4	999.34	+26	25,957.30	Snake venom metalloprotease	23433.41 (non-glycosylated)	D_polylepis_T3990_T0439	SVMP
	16.8	1055.34	7+	7377.36	3SIM3_DENAN (Muscarinic toxin 3) *Dendroaspis angusticeps*	7392.54	D_polylepis_T2320	3FTx
	16.8	1055.05	36+	37,947.16	Snake venom metalloproteinase	27,106.10 (non-glycosylated)	D_polylepis_T0167	SVMP
	17.7	1081.83	6+	6481.97	Three finger toxins	N.A.	D_polylepis_T2331	3FTx
*Dendroaspis angusticeps*	15.1	965.14	7+	6745.91	3SE2_DENAN (Fasciculin-2) *Dendroaspis angusticeps*	6744.96	D_angusticeps_T1380_T2642	3FTx
	15.5	971.99	7+	6793.93	3SE1_DENAN (Fasciculin-1) *Dendroaspis angusticeps*	6793.98	D_angusticeps_T2737	3FTx
	16.1	943.44	7+	6593.05	Three-finger toxin	N.A.	D_angusticeps_T1011	3FTx
	16.4	945.44	7+	6607.07	VKTHE_DENAN (Kunitz-type serine protease inhibitor long epsilon-dendrotoxin His55) *Dendroaspis angusticeps*	6609.17	D_angusticeps_T3547	Kunitz-type serine protease
	17.1	1073.63	7+	7504.03	3SI1B_DENAN (Rho-elapitoxin-Da1b) *Dendroaspis angusticeps*	7507.48	D_angusticeps_T4405	3FTx
		1048.76	7+	7330.29	3NOJ_DENAN (Toxin Tx7335) *Dendroaspis angusticeps*	7330.33	D_angusticeps_T3777	3FTx
	17.1	1045.05	59+	61,599.77	Snake venom metalloproteinase	62,965.82	D_angusticeps_T0082	SVMP
	18.2	951.35	7+	6648.35	3SA5_NAJKA (Cytotoxin 5) *Naja kaouthia*	6846.95	N.A.	3FTx
*Naja naja*	15.3	1116.36	7+	7803.47	3L22_NAJNA (Long neurotoxin 2) *Naja naja*	7805.48	N.A.	3FTx
	15.7	1118.79	7+	7819.46	3L23_NAJNA (Long neurotoxin 3) *Naja naja*	7817.54	N.A.	3FTx
	16.3	1663.70	8+	13,293.52	PA2B3_NAJMO (Basic phospholipase A_2_ CM-III) *Naja mossambica*	13,288.82	N.A.	PLA_2_
		1678.07	8+	13,408.51	PA2A2_NAJME (Acidic phospholipase A_2_ DE-II) *Naja melanoleuca*	13,413.07	N.A.	PLA_2_
	16.4	972.19	7+	6795.27	3SA4_NAJHA (Cytotoxin 4) *Naja haje*	6794.58	N.A.	3FTx
	16.5	1482.07	9+	13,321.59	PA2A2_NAJNA (Acidic phospholipase A_2_ 2) *Naja naja*	13,322.64	N.A.	PLA_2_
		1474.18	9+	13,249.27	PA2B4_NAJNG (Basic phospholipase A_2_) *Naja nigricollis*	13,245.22	N_naja_T0142_T1042_T2728_T2640_ T2414_T1496_T0668_T2421	PLA_2_
		1447.12	9+	13,007.02	NGFV_NAJNA (Venom nerve growth factor) *Naja naja*	13,008.10	N.A.	vNGF
		1681.58	8+	13,435.58	Phospholipase A_2_	N.A.	N.A.	PLA_2_
	16.6	1649.45	8+	13,179.51	PA2A_NAJAT (Acidic phospholipase A_2_ natratoxin) *Naja atra*	13,174.25	N.A.	PLA_2_
		1672.58	8+	13,364.56	Phospholipase A_2_	N.A.	N.A.	PLA_2_
	16.6	2217.76	12+	26,598.04	Snake venom metalloproteinase	N.A.	N.A.	SVMP
	17.2	1001.78	7+	7002.44	3SOFL_NAJNA (Cytotoxin like basic protein) *Naja naja*	7006.56	N_naja_T0917	3FTx
	17.4	965.91	7+	6750.33	3SAA_NAJNA (Cytotoxin 10) *Naja naja* 3SA2_NAJNA (Cytotoxin 2) *Naja naja*	6751.34 6750.36	N_naja_T2400_T1263_T0382_T2324	3FTx 3FTx
		963.33	7+	6733.32	3SA3_NAJNA (Cytotoxin 3) *Naja naja*	6732.34	N.A.	3FTx
	17.6	969.7+	7+	6778.33	3SA1_NAJNA (Cytotoxin 1) *Naja naja* 3SA7_NAJNA (Cytotoxin 7) *Naja naja* 3SA8_NAJNA (Cytotoxin 8) *Naja naja*	6778.32 6779.30 6780.28	N_naja_T0481	3FTx 3FTx 3FTx
	18.3	1113.05	6+	6669.27	3SA9_NAJNA (Cytotoxin 9) *Naja naja*	6661.77	N_naja_T2420_T2418_T2687_T1672	3FTx
*Naja pallida*	12.5	970.74	7+	6785.08	3SA4_NAJHA (Cytotoxin 4) *Naja haje*	6789.37	N_pallida_T1076 N_pallida_T0738	3FTx
	14.9	987.35	7+	6901.42	Three finger toxins	N.A.	N.A.	3FTx
	16.2	1478.76	9+	13,290.79	(Basic phospholipase A_2_) *Naja pallida*	13,266.83	N_pallida_T0443	PLA_2_
	16.4	1496.20	9+	13,205.74	Phospholipase A_2_	N.A.	N.A.	PLA_2_
		1666.72	8+	13,320.73	PA2B_NAJPA (Basic phospholipase A_2_ nigexine) *Naja pallida*	13,316.93	N.A.	PLA_2_
		963.19	7+	6732.29	Three finger toxins	6731.85	N_pallida_T2193_T1690	3FTx
	17.3	994.06	7+	6948.36	3S1CB_NAJNA (Cobrotoxin homolog) *Naja naja*	6943.98	N.A.	3FTx
	17.5	970.34	7+	6782.34	3S11_NAJPA (Short neurotoxin 1) *Naja pallida*	6782.10	N_pallida_T0954	3FTx
	17.8	984.77	7+	6883.39	3SA3_NAJMO (Cytotoxin 3) *Naja mossambica*	6881.42	N_pallida_T1038_T1323	3FTx
	18.2	975.05	7+	6815.31	3SA1_NAJPA (Cytotoxin 1) *Naja pallida*	6814.31	N_pallida_T1011_T2149_T1010_T0889	3FTx
*Naja nigricollis*	15.6	1036.75	7+	7247.19	Three-finger toxin	N.A.	N.A.	3FTx
	16.1	1473.65	9+	13,245.77	PA2B4_NAJNG (Phospholipase A2 Basic) *Naja nigricollis*	13,244.90	N_nigricollis_T2086_T1848_T3198	PLA_2_
	16.8	1477.42	9+	13,279.66	Acidic phospholipase A_2_	13,279.08	N_nigricollis_T3199_T0053	PLA_2_
	17.0	959.33	7+	6705.27	3SA4_NAJMO (Cytotoxin 4) *Naja mossambica*	6702.34	N.A.	3FTx
		975.33	7+	6816.29	3SA1_NAJPA (Cytotoxin 1) *Naja pallida*	6814.31	N_nigricollis_VG_T3171_T1648	3FTx
	17.2	965.90	7+	6751.26	3SA9_NAJNA (Cytotoxin 9) *Naja naja*	6570.36	N.A.	3FTx
		1656.97	9+	13,167.58	PA2A1_NAJMO (Acidic phospholipase A_2_ CM-I	13,195.75	N.A.	PLA_2_
	17.4	956.17	7+	6683.20	3SAN_NAJNG (Naniproin) *Naja nigricollis*	6682.41	N_nigricollis_VG_T2771	3FTx
	17.7	947.48	7+	6811.32	3SA5_NAJAT (Cytotoxin 5) *Naja atra*	6810.35	N.A.	3FTx
	18.0	984.77	7+	6883.38	3SA3_NAJMO (Cytotoxin 3) *Naja mossambica*	6881.42	N.A.	3FTx
	18.4	975.05	7+	6815.31	3SA1_NAJMO (Cytotoxin 1) *Naja mossambica*	6813.33	N_nigricollis_VG_T1821_T1178	3FTx
*Naja haje*	14.4	963.21	8+	7692.63	Three finger toxins	N.A.	N.A.	3FTx
	16.1	1031.16	7+	7208.09	3SI3_NAJMO (Short neurotoxin 3) *Naja mossambica*	7210.58	N.A.	3FTx
	16.5	1552.38	9+	13,963.36	PA2A3_NAJSG (Acidic phospholipase A_2_ 3) *Naja sagitifera*	13,955.23	N.A.	PLA_2_
	16.9	981.89	7+	6863.21	3SA2_NAJME (Cytotoxin 2) *Naja melanoleuca*	6863.54	N_haje_T2831_T1704_T3906_T3905_ T0971	3FTx
	17.2	967.31	7+	6761.18	3SA5_NAJHH (Cytotoxin 5) *Naja haje*	6765.18	N.A.	3FTx
	17.6	974.73	7+	6813.29	3SA1_NAJMO (Cytotoxin 1) *Naja mossambica*	6813.33	N.A.	3FTx
	17.9	979.32	7+	6845.24	3SA2_NAJHA (Cytotoxin 2) *Naja haje* 3SA5_NAJHA (Cytotoxin 5) *Naja haje* 3SA6_NAJHA (Cytotoxin 6) *Naja haje* 3SA7_NAJHA (Cytotoxin 7) *Naja haje*	6845.24 6843.27 6844.25 6843.27	N.A.	3FTx 3FTx 3FTx 3FTx
*Hemachatus haemachatus*	15.3	1014.45	7+	7091.10	3SUB_DENAN (Muscarinic toxin) *Dendroaspis angusticeps*	7092.35	N.A.	3FTx
	15.8	1501.53	9+	13,498.76	PA2B1_HEMHA (Basic phospholipase A2 DE-1) *Hemachatus haemachatus*	13,495.94	H_haemachatus_T1146	PLA_2_
	15.8	1031.22	7+	7208.50	Three finger toxins	N.A.	N.A.	3FTx
	16.4	971.21	7+	6788.43	3S11_NAJPA (Short neurotoxin 1) *Naja pallida* 3SA4_NAJHA (Cytotoxin 4) *Naja haje* 3SB2_HEMHA (Cytotoxin 2) *Hemachatus haemachatus*	6782.10 6789.37 6787.44	H_haemachatus_T1235	3FTx 3FTx 3FTx
	16.7	977.50	7+	6832.45	3S11_NAJHA (Short neurotoxin 1) *Naja haje* 3SBH_HEMHA (Three-finger hemachatoxin) *Hemachatus haemachatus* 3SB1_HEMHA (Cytotoxin 1) *Hemachatus haemachatus*	6831.05 6381.45 6831.45	H_haemachatus_T1274_T1588_T1866_ T1092_T1440_T1175	3FTx 3FTx 3FTx
	18.0	972.34	7+	6795.35	3SA8_NAJHA (Cytotoxin 8) *Naja haje*	6799.32	N.A.	3FTx
	18.2	969.91	7+	6779.36	3SB3_HEMHA (Cytotoxin 3) *Hemachatus haemachatus*	6780.32	N.A.	3FTx

## Data Availability

The underpinning raw MS data of seven elapid venom species, MGF files, and LC-UV raw chromatogram can be found in FigShare using the following links. MGF Files of cobra venom DOI: doi.org/10.6084/m9.figshare.19397903. MGF Files of mamba venom DOI: doi.org/10.6084/m9.figshare.19397852. LC-UV raw chromatogram DOI: doi.org/10.6084/m9.figshare.19447583.

## Supplementary Materials

The following supporting information can be downloaded at: https://www.mdpi.com/article/10.3390/toxins14110736/s1, Supplementary Data contains: (a) raw mass spectrometry data of seven elapid venom species. The raw data were generated from Bruker mass spectrometer and can be accessed using Compass Data Analysis 5.0 software. (b) The Mascot Generic Files (MGF) were created from deconvoluting the raw mass spectrometry data using nanoLC–MS/MS. (c) an LC-UV raw chromatogram data that was generated using Shimadzu Lab Solution software. Supplementary Figures consist of figures that are not shown in the main text: Supplementary Figure S1. Elapid venoms demonstrate varying degrees of anticoagulant activity. Supplementary Figure S2. The PLA2 inhibitor varespladib, but not the SVMP inhibitor marimastat, cause significant reductions in anticoagulant activity induced by cobra venom. Supplementary Figure S3. The anticoagulant curves of venom with the addition of the PLA2 inhibitor varespladib show a reduction in the anticoagulant activity of cobra venoms with increasing doses of varespladib. Supplementary Figure S4. The coagulation curves of venom with the addition of the SVMP inhibitor marimastat show no reduction in the anticoagulant activity of cobra venoms but do inhibit the procoagulant toxins *of Hemachatus haemachatus*, causing the venom to become anticoagulant. Supplementary Figures S5–S11 Duplication results of superimposed pro- and anticoagulation bioassay chromatogram of *Dendroaspis angusticeps, D. angusticeps, Naja naja, Naja pallida, Naja nigricollis, Naja haje* and, *Hemachatus haemachatus* venom respectively. Figures S12–S18 Detailed results of proteomics searches using Mascot software against the Species-specific database and Swiss-Prot database to identify anticoagulant venom proteins of *Dendroaspis angusticeps, D. angusticeps, Naja naja, Naja pallida, Naja nigricollis, Naja haje* and, *Hemachatus haemachatus* venom respectively.
