# Supplementary material for: A Combined Bioassay and Nanofractionation Approach to Investigate the Anticoagulant Toxins of Mamba and Cobra Venoms and Their Inhibition by Varespladib
Source: Toxins (Basel). 2022 Oct 27;14(11):736. doi: 10.3390/toxins14110736 (PMC9695013; doi:10.3390/toxins14110736)
Supplement: Supplementary file 1 [file toxins-14-00736-s001.zip › toxins-1946944-supplementary.pdf]

toxins

MDPI

A combined bioassay and nanofractionation approach to investigate the anticoagulant toxins of mamba and cobra venoms and their inhibition by Varespladib

Arif Arrahman[1,2,3], Taline D. Kazandjian[4], Kristina B.M. Still[1,2], Julien Slagboom[1,2], Govert W. Somsen[1,2], Freek J. Vonk[1,5], Nicholas R. Casewell[4] and Jeroen Kool[1,2,*]

[1]Division of Bioanalytical Chemistry, Department of Chemistry and Pharmaceutical Sciences, Faculty of Sciences, Amsterdam Institute of Molecular and Life Sciences (AIMMS), Vrije Universiteit Amsterdam, Amsterdam, The Netherlands.

[2]Centre for Analytical Sciences Amsterdam (CASA), Amsterdam, The Netherlands.

[3]Faculty of Pharmacy, Universitas Indonesia, Depok, Indonesia.

[4]Centre for Snakebite Research and Interventions, Liverpool School of Tropical Medicine, Liverpool, United Kingdom.

[5]Naturalis Biodiversity Center, Leiden, The Netherlands.

*** Correspondence:**
Jeroen Kool
J.Kool@vu.nl

Supplementary Material

1 Supplementary Data

The supplementary data of this study consists of (a) raw mass spectrometry data of seven elapid venom species. The raw data was generated from Bruker mass spectrometer and can be accessed using Compass Data Analysis 5.0 software. (b) The Mascot Generic Files (MGF) that were created from deconvoluting the raw mass spectrometry data using nanoLC-MS/MS and Data Analysis program were set with the following parameters: charge deconvolution for peptides/small molecules (1) adduct ions: positive (+H); (2) deconvolute MS and MS(n) spectra; (3) abundance cutoff: 10%; (4) maximum charge: 10. The MGF was named after the retention time of particular fractions and related species. Both supplementary data were available and can be used for other research purposes. (c) an LC-UV raw chromatogram data that was generated using Shimadzu Lab Solution software.

2 Supplementary Figures and Tables

The reconstructed coagulation bioassay chromatograms show the effect of individual venom proteins for *Dendroaspis polylepis* (Tanzania), *Dendroaspis angusticeps* (Tanzania), *Naja naja* (captive-bred), *Naja pallida* (Tanzania), *Naja nigricollis* (Tanzania), *Naja haje* (Uganda), and *Hemachatus haemachatus* (South Africa) venom after nanofractionation at different concentrations are shown in Supplementary Figures 5-11. In this study, each coagulation bioassay was performed at least in

duplicate following nanofractionation to assess reproducibility. In each figure, the bioassay chromatogram **A** consists of UV Chromatogram at the top. The middle series of superimposed bioassay chromatograms represent the anticoagulant and the lower series represents coagulation analyses for different venom concentrations. In this study, there was no procoagulation activity detected in the samples. The highest venom concentration was 1.0 mg/mL used in this experiment. When the venom was analysed at 1.0 mg/mL, a broad negative peak (full anticoagulation) was observed. This broad negative peak most probably represents the bioactivity of multiple closely eluting peaks from several proteins involved in the anticoagulation activity. The bioassay chromatogram **B** at the middle series of superimposed bioassay chromatograms represent the inhibition of anticoagulant activity in the presence of varespladib with concentrations ranging from 20 µM to 0.032 µM. The bioassay chromatogram **C** at the middle series of superimposed bioassay chromatograms represents the effect on anticoagulant activity of marimastat with concentrations ranging from 20 µM to 32 nM.

2.1 Supplementary Figures

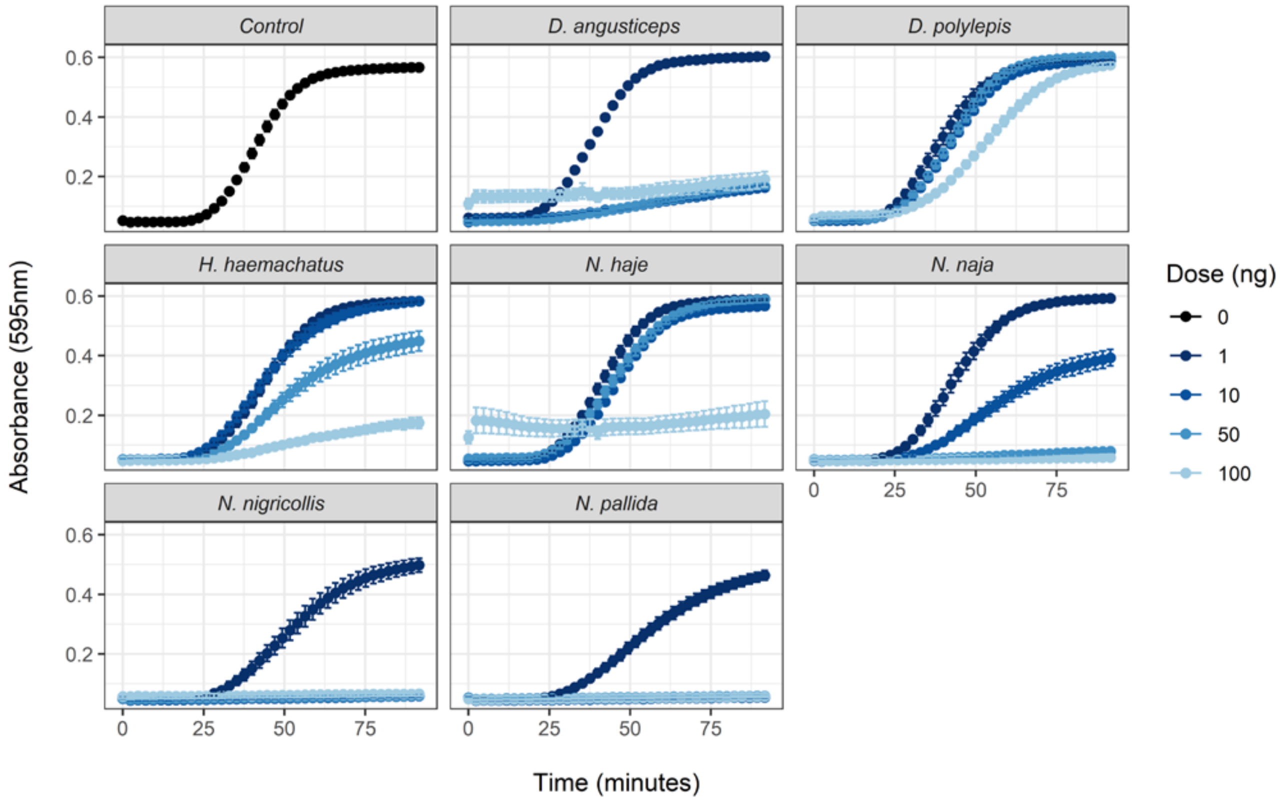

Supplementary Figure S1. Elapid venoms demonstrate varying degrees of anticoagulant activity. Coagulation is measured here as the mean absorbance of light through the bovine plasma at the 595 nm wavelength. Error bars represent the standard error of the mean (SEM) of 3-7 replicates (some sample wells needed to be removed due to disruption in readings caused by bubbles).

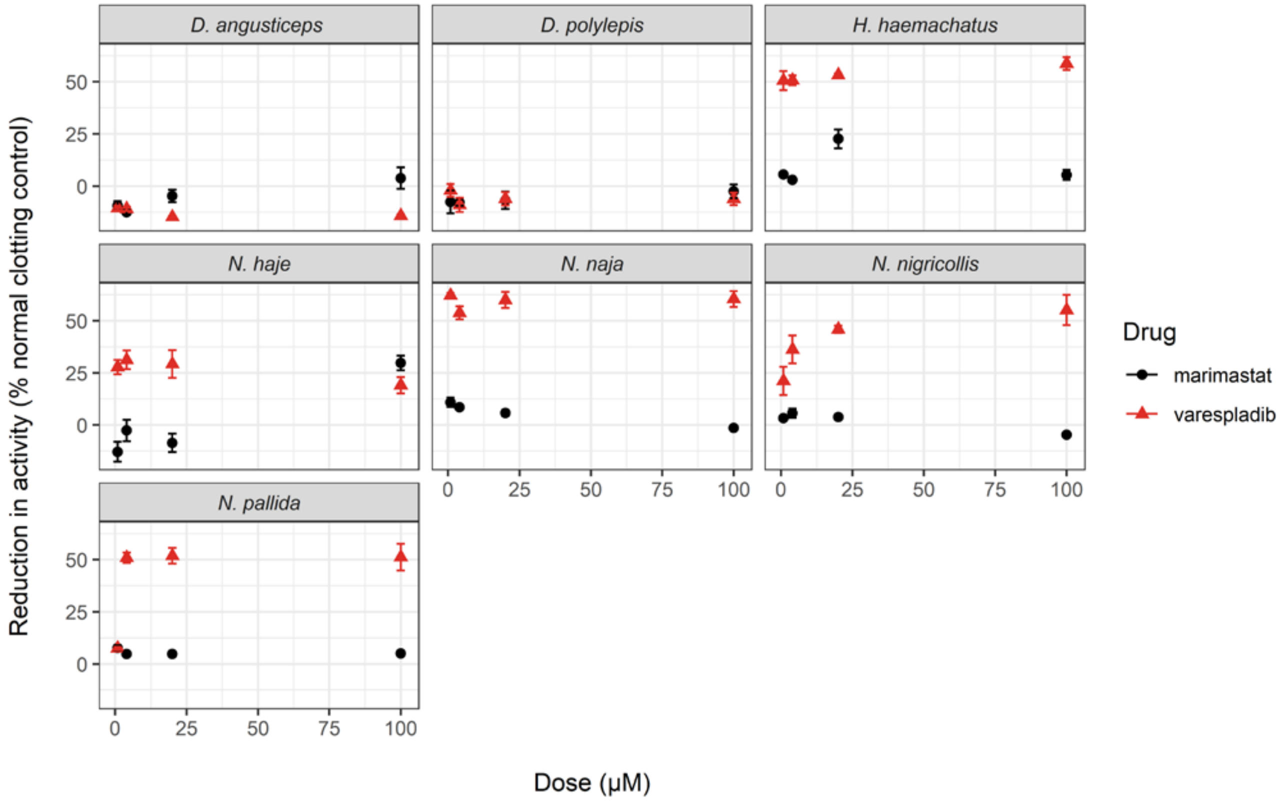

Supplementary Figure S2. The PLA_2 inhibitor varespladib, but not the SVMP inhibitor marimastat, cause significant reductions in anticoagulant activity induced by cobra, but not mamba, venoms, with the exception of *Naja haje*, in which the highest dose causes reduction in activity comparable to varespladib. Reduction in activity was measured as the mean area under the curve (AUC) of each inhibitor + venom sample (100 ng) in the relevant clotting period (22-71minutes), standardised by the mean AUC of the negative control in the same period, minus the standardised crude venom AUC. Error bars represent the standard error of the mean (SEM) of 3-4 replicates (some sample wells needed to be removed due to disruption in readings caused by bubbles).

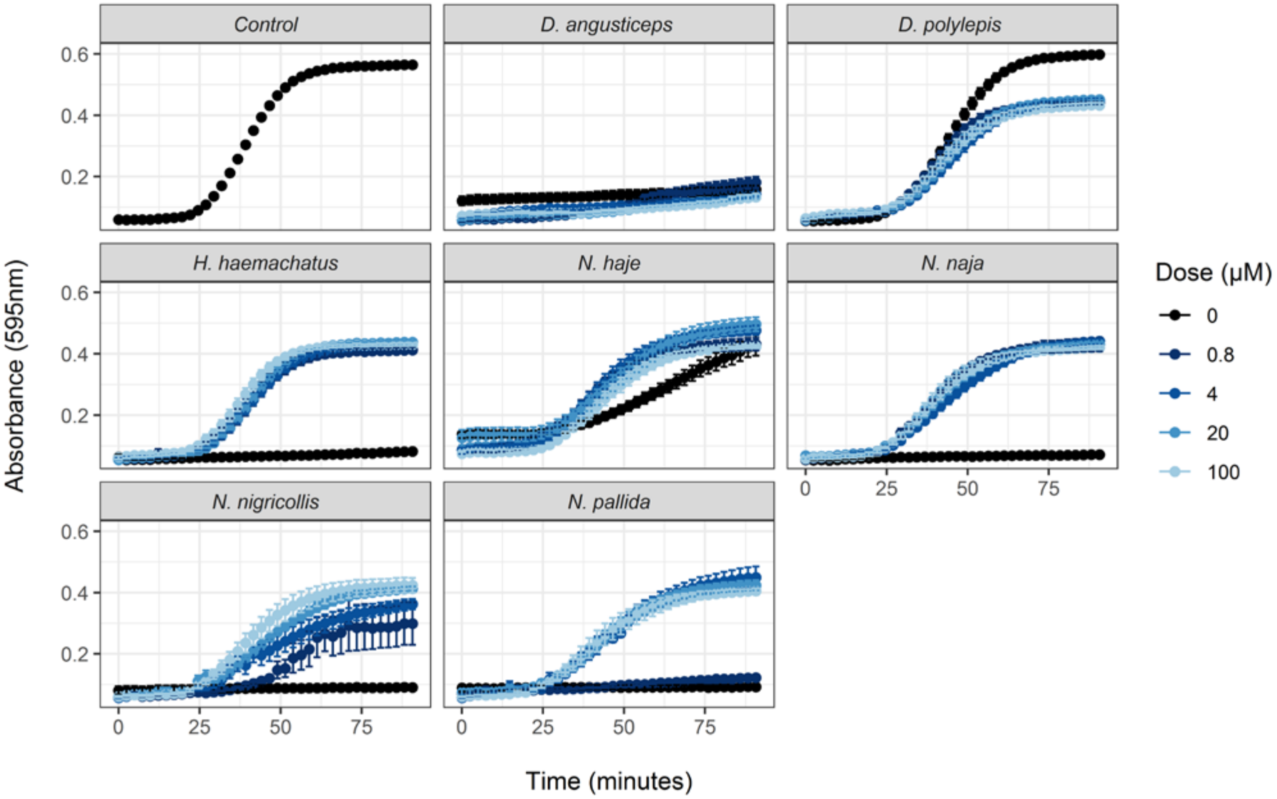

Supplementary Figure S3. The coagulation curves of 100 ng venom with the addition of the PLA_2 inhibitor varespladib show a reduction in the anticoagulant activity of cobra, but not mamba, venoms with all doses of varespladib. Coagulation is measured here as the mean absorbance of light through the bovine plasma at the 595 nm wavelength. Error bars represent the standard error of the mean (SEM) of 3-7 replicates (some sample wells needed to be removed due to disruption in readings caused by bubbles).

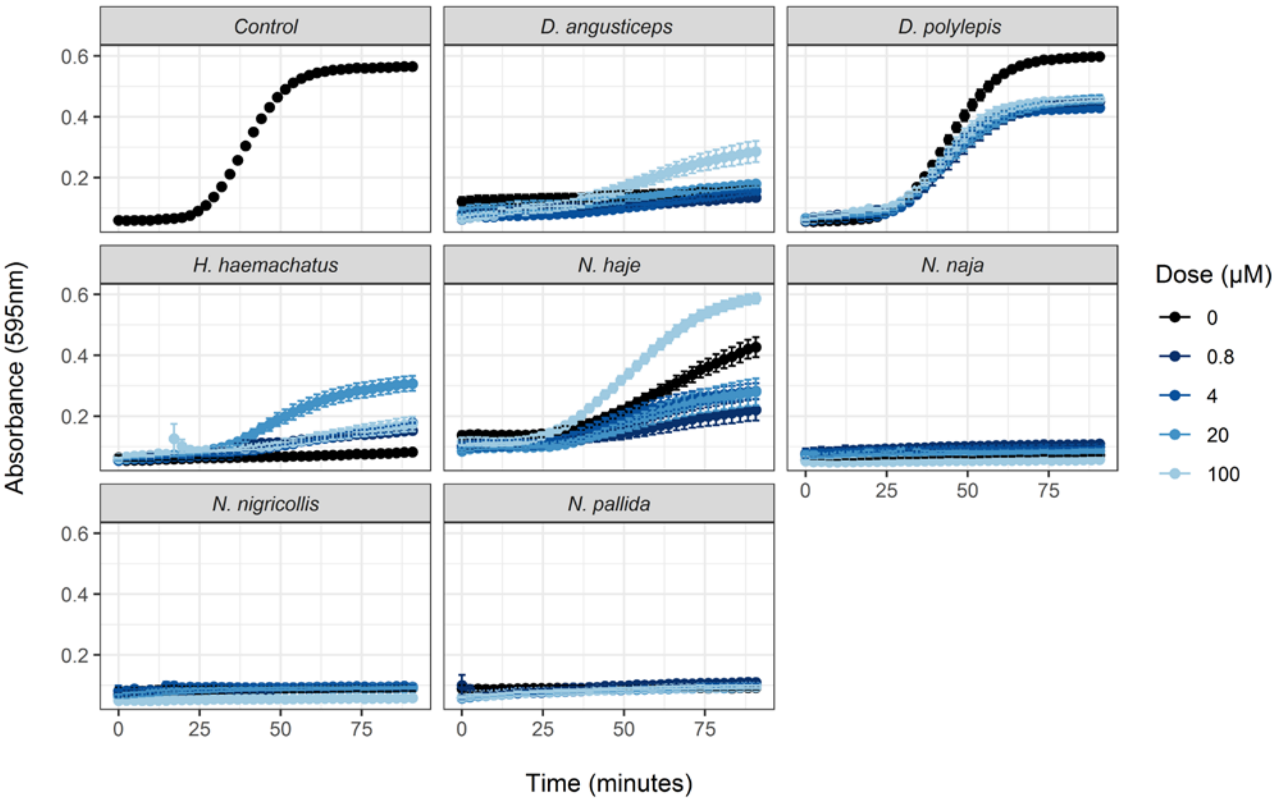

Supplementary Figure S4. The coagulation curves of 100 ng venom with the addition of the SVMP inhibitor marimastat show no significant reductions in the anticoagulant activity of the venoms of African elapids, with the exception of *Hemachatus haemachatus* and *Naja haje*. Coagulation is measured here as the mean absorbance of light through the bovine plasma at the 595 nm wavelength. Error bars represent the standard error of the mean (SEM) of 3-4 replicates.

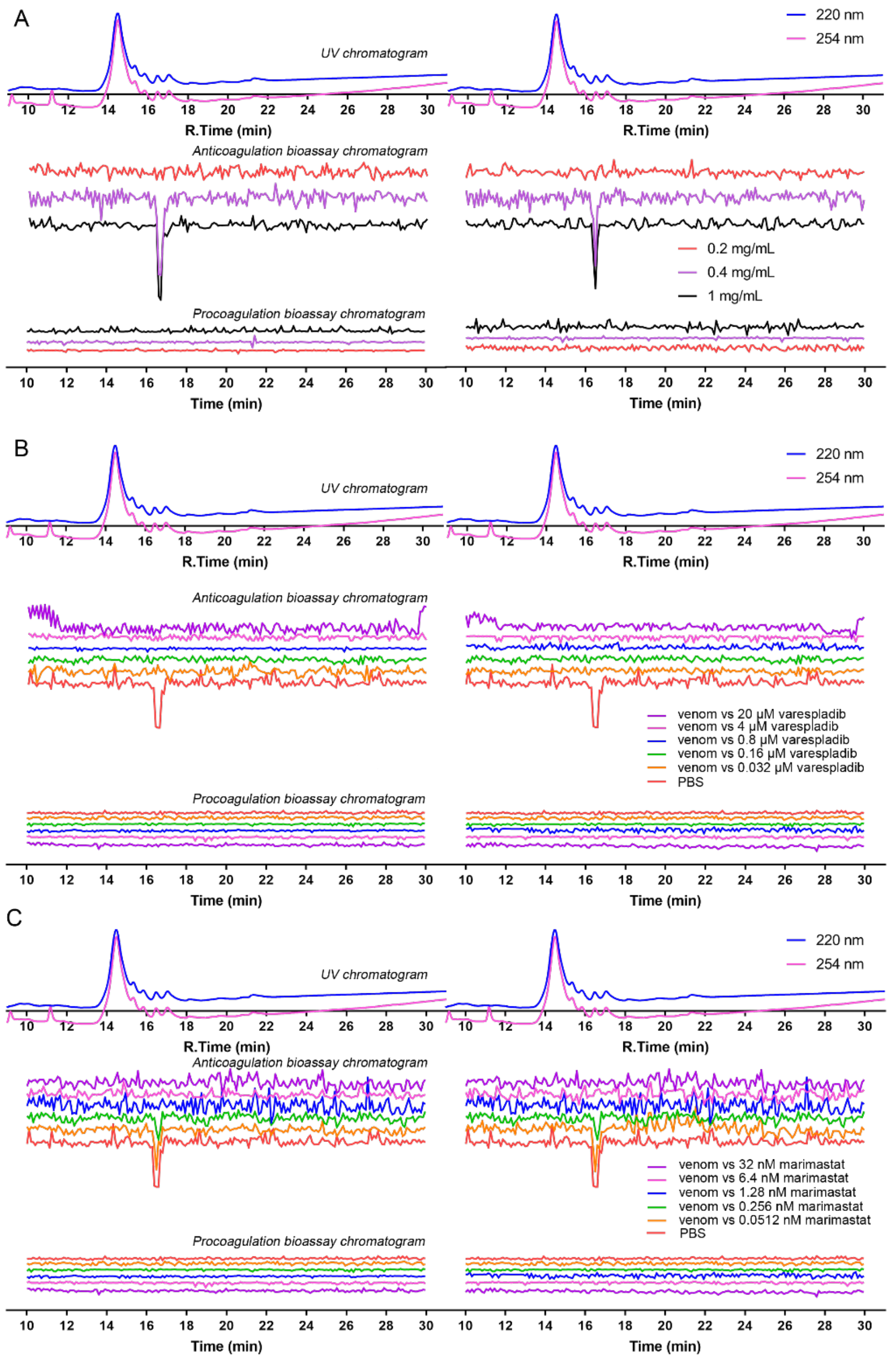

Supplementary Figure S5. Duplication results of superimposed pro- and anticoagulation bioassay chromatogram of *Dendroaspis polylepis* venom. (A) the duplicate data showed results from analyses of serial diluted *Dendroaspis polylepis* venom ranging from 1 mg/mL to 0.2 mg/mL (50 µL per injection). (B) bioassay chromatogram at 1 mg/mL concentration venom (50 µL per injection) in the presence of different concentrations of varespladib. Varespladib effectively inhibited anticoagulant venom effects in a dose-dependent manner with inhibitor concentrations ranging from 20 µM to 0.16 µM. (C) bioassay chromatogram at 1 mg/mL concentration venom (50 µL per injection) in the presence of different concentrations of marimastat. Marimastat effectively inhibited the anticoagulant

venom effect in a dose-dependent manner with inhibitor concentrations ranging from 32 nM to 0.0512 nM.

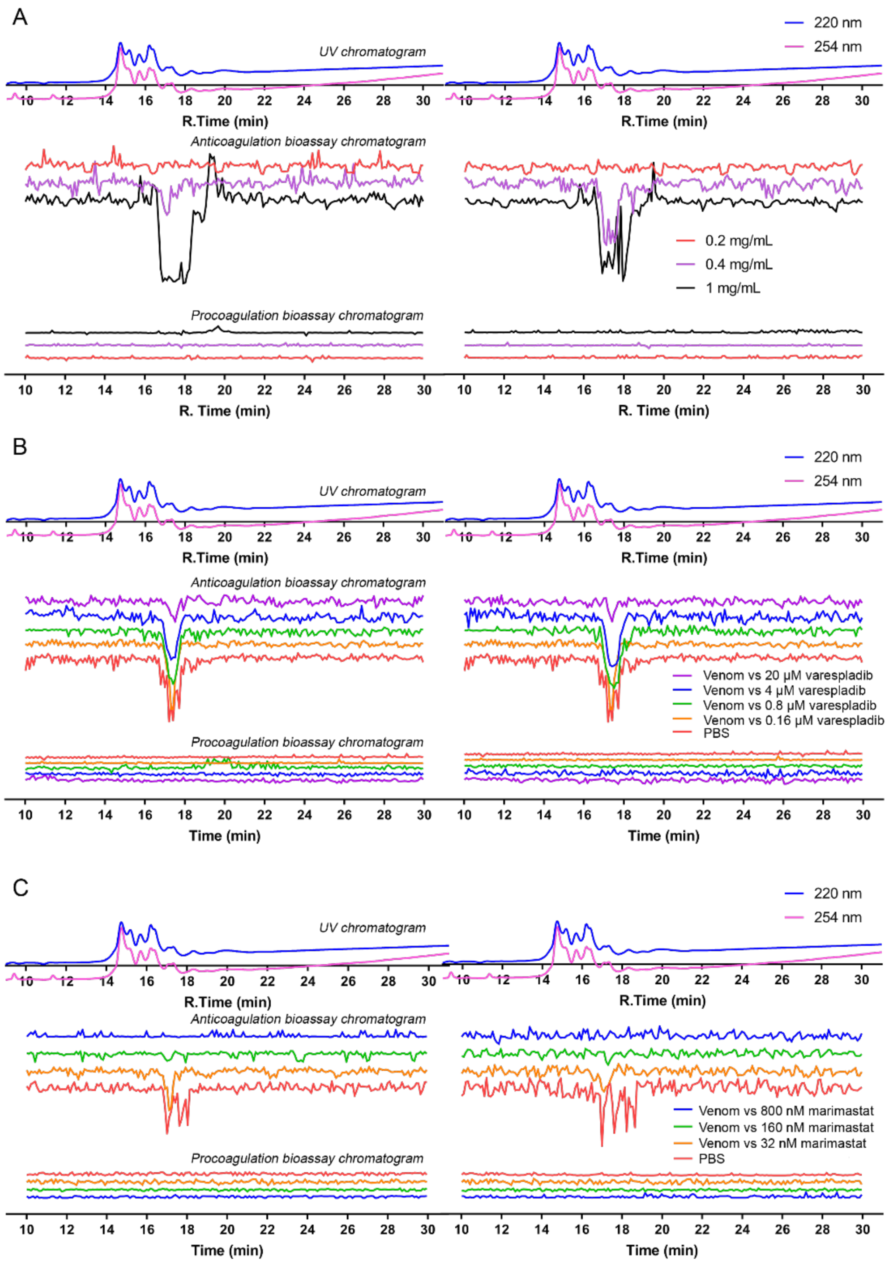

Supplementary Figure S6. Duplication results of superimposed pro- and anticoagulation bioassay chromatogram of *Dendroaspis angusticeps* venom. (A) the duplicate data showed results from analyses of serial diluted *Dendroaspis angusticeps* venom ranging from 1 mg/mL to 0.2 mg/mL (50 µL per injection). (B) bioassay chromatogram at 1 mg/mL concentration venom (50 µL per injection)

in the presence of different concentrations of varespladib. Varespladib effectively inhibited anticoagulant venom effects in a dose-dependent manner with inhibitor concentrations ranging from 20 µM to 0.16 µM. (C) bioassay chromatogram at 1 mg/mL concentration venom (50 µL per injection) in the presence of different concentrations of marimastat. Marimastat effectively inhibited the anticoagulant venom effect in a dose-dependent manner with inhibitor concentrations ranging from 800 nM to 32 nM.

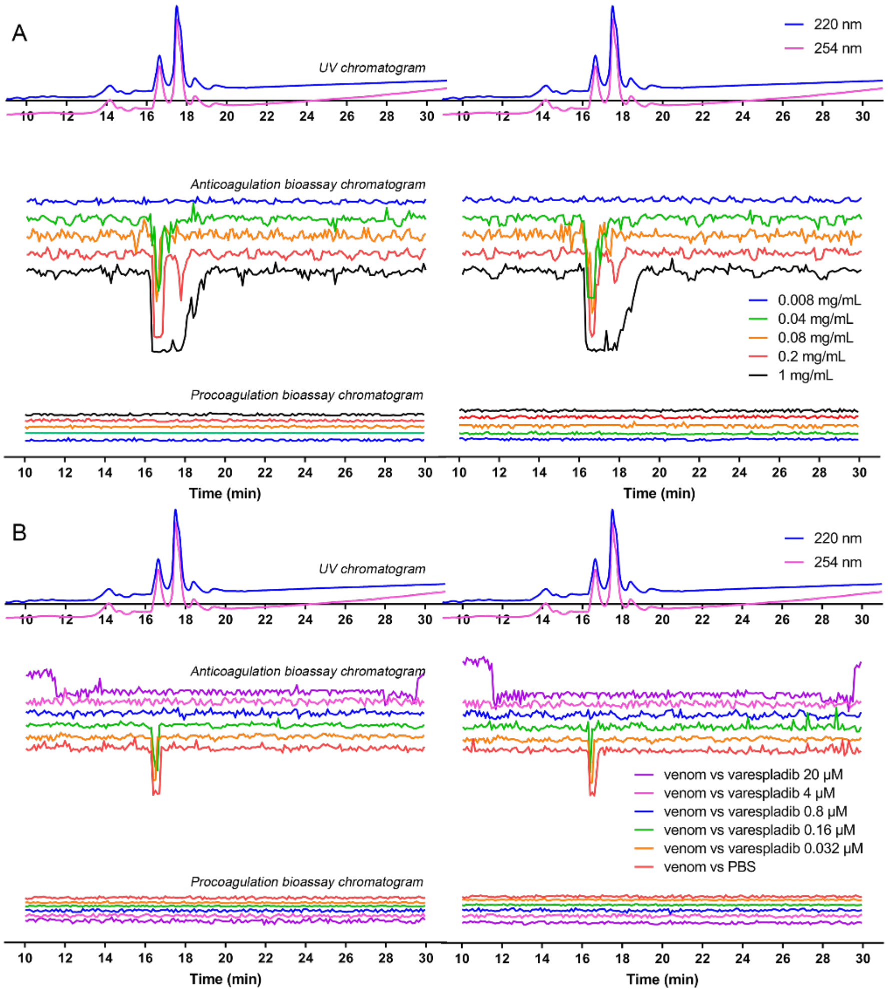

Supplementary Figure S7. Duplication results of superimposed pro- and anticoagulation bioassay chromatogram of *Naja naja* venom. (A) the duplicate data showed results from analyses of serial diluted *Naja naja* venom ranging from 1 mg/mL to 0.2 mg/mL (50 µL per injection). (B) bioassay chromatogram at 0.2 mg/mL concentration venom (50 µL per injection) in the presence of different concentration varespladib. Varespladib effectively inhibited anticoagulant venom effects in a dose-dependent manner with inhibitor concentrations ranging from 20 µM to 0.032 µM.

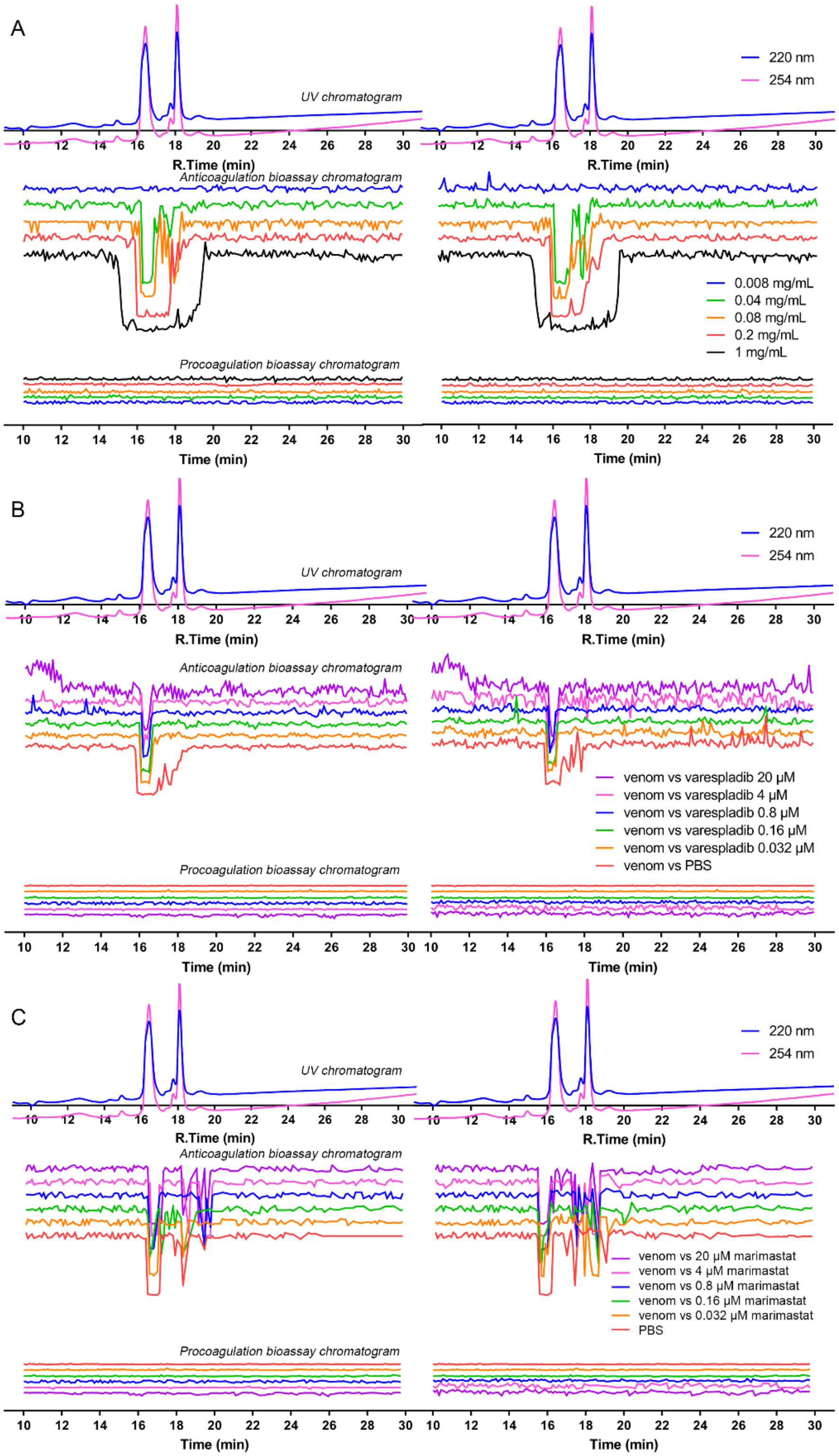

Supplementary Figure S8. Duplication results of superimposed pro- and anticoagulation bioassay chromatogram of *Naja pallida* venom. (A) the duplicate data showed results from analyses of serial diluted *Naja pallida* venom ranging from 1 mg/mL to 0.008 mg/mL (50 µL per injection). (B) bioassay chromatogram at 0.2 mg/mL concentration venom (50 µL per injection) in the presence of

different concentration varespladib. Varespladib effectively inhibited anticoagulant venom effects in a dose-dependent manner with inhibitor concentrations ranging from 20 µM to 0.032 µM. (C) bioassay chromatogram at 1 mg/mL concentration venom (50 µL per injection) in the presence of different concentrations of marimastat. Marimastat has no anticoagulant inhibition activity on elapid venom.

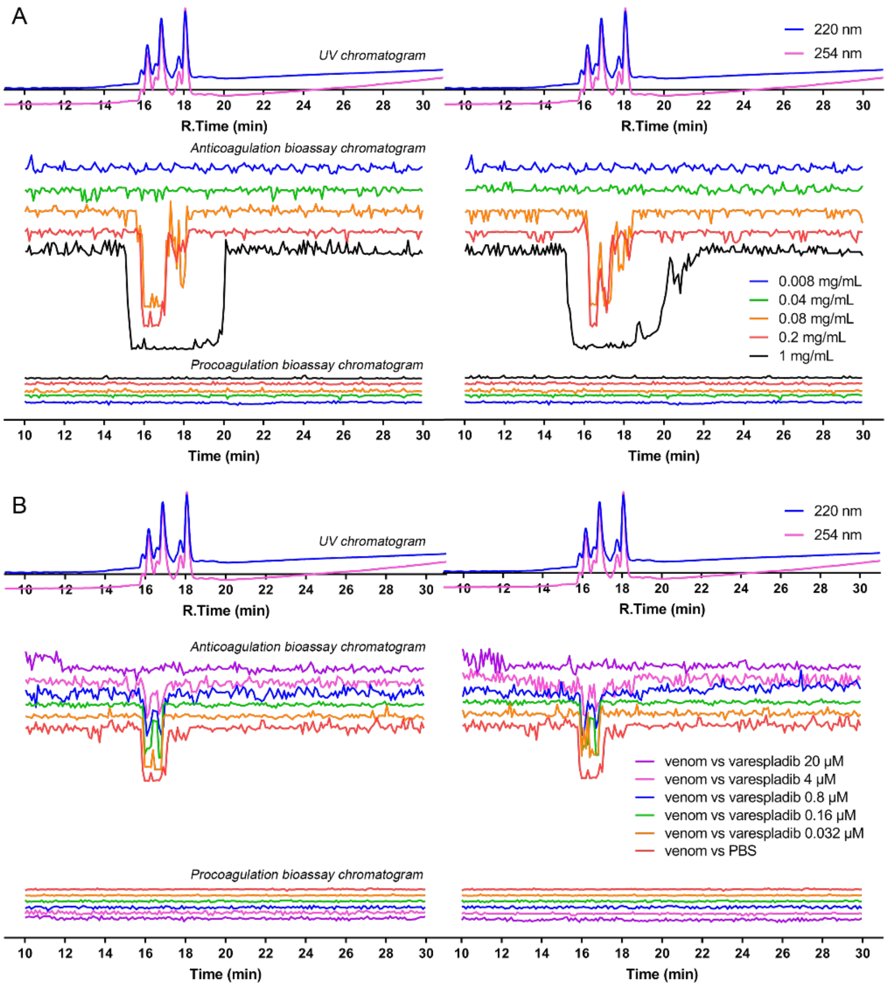

Supplementary Figure S9. Duplication results of superimposed pro- and anticoagulation bioassay chromatogram of *Naja nigricollis* venom. (A) the duplicate data showed results from analyses of serial diluted *Naja nigricollis* venom ranging from 1 mg/mL to 0.008 mg/mL (50 µL per injection). (B) bioassay chromatogram at 0.2 mg/mL concentration venom (50 µL per injection) in the presence of different concentration varespladib. Varespladib effectively inhibited anticoagulant venom effects in a dose-dependent manner with inhibitor concentrations ranging from 20 µM to 0.032 µM.

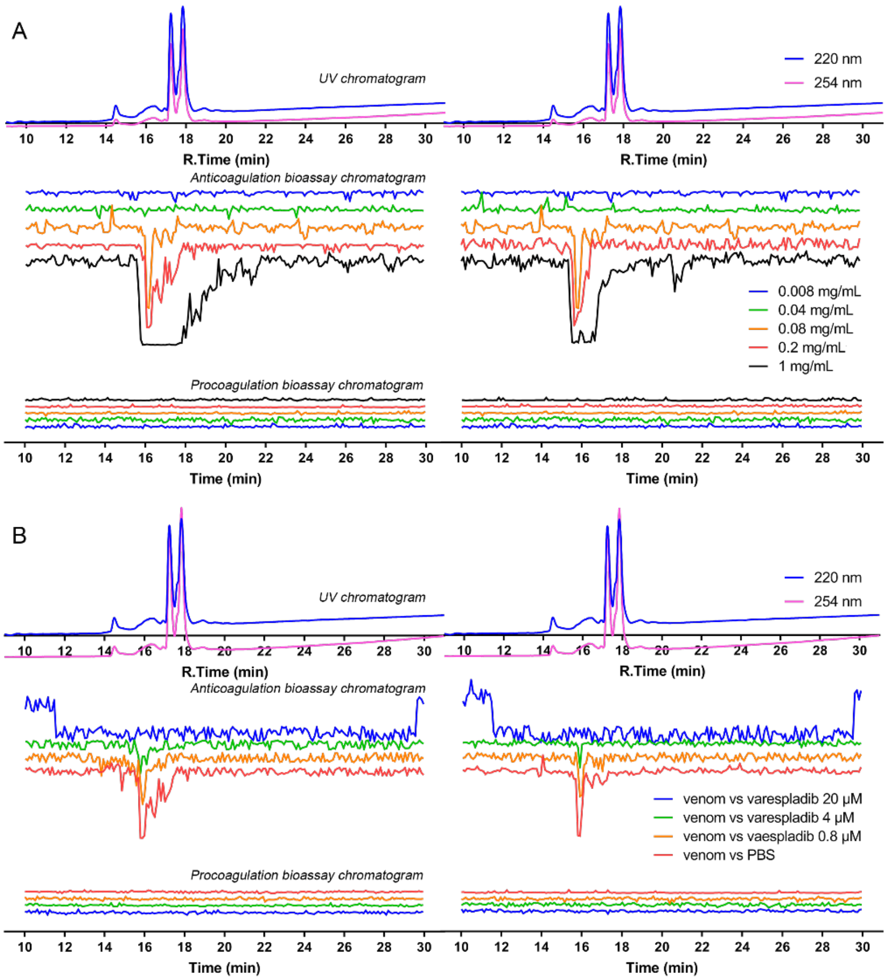

Supplementary Figure S10. Duplication results of superimposed pro- and anticoagulation bioassay chromatogram of *Naja haje* venom. (A) the duplicate data showed results from analyses of serial diluted *Naja haje* venom ranging from 1 mg/mL to 0.008 mg/mL (50 µL per injection). (B) bioassay chromatogram at 0.2 mg/mL concentration venom (50 µL per injection) in the presence of different concentration varespladib. Varespladib effectively inhibited anticoagulant venom effects in a dose-dependent manner with inhibitor concentrations ranging from 20 µM to 0.8 µM.

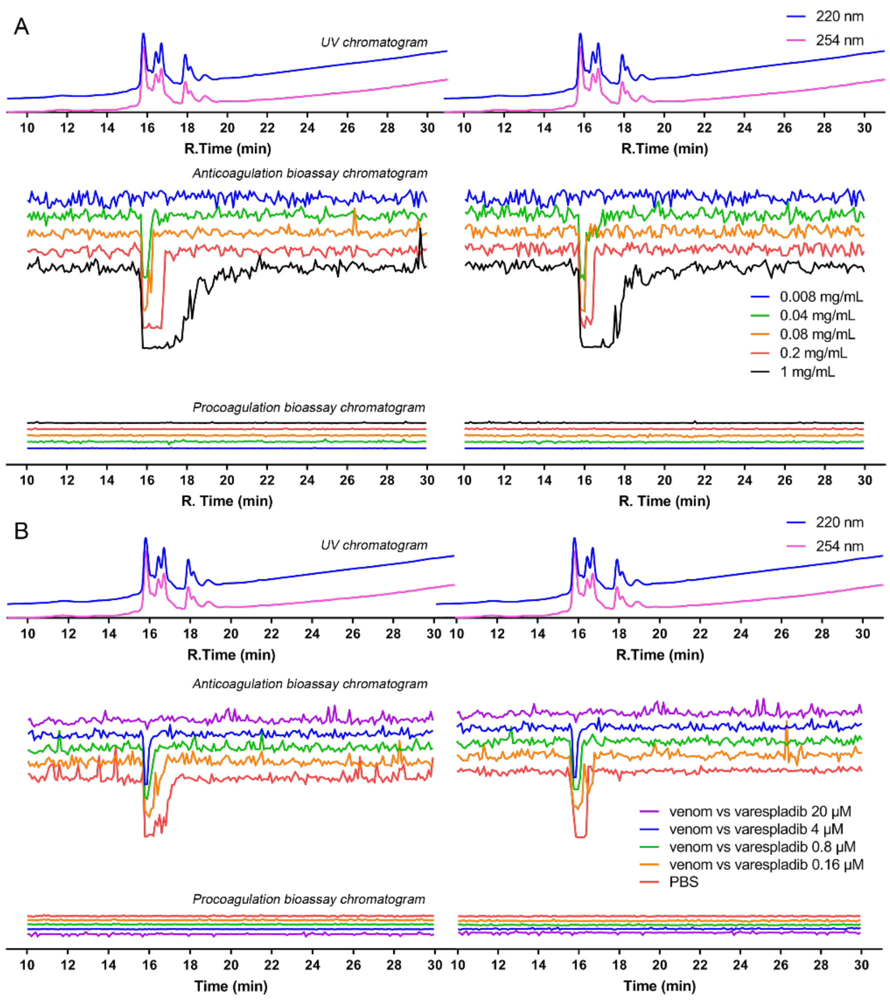

Supplementary Figure S11. Duplication results of superimposed pro- and anticoagulation bioassay chromatogram of *Hemachatus haemachatus* venom. (A) the duplicate data showed results from analyses of serial diluted *Hemachatus haemachatus* venom ranging from 1 mg/mL to 0.008 mg/mL (50 µL per injection). (B) bioassay chromatogram at 0.2 mg/mL concentration venom (50 µL per injection) in the presence of different concentration varespladib. Varespladib effectively inhibited anticoagulant venom effects in a dose-dependent manner with inhibitor concentrations ranging from 20 µM to 0.16 µM.

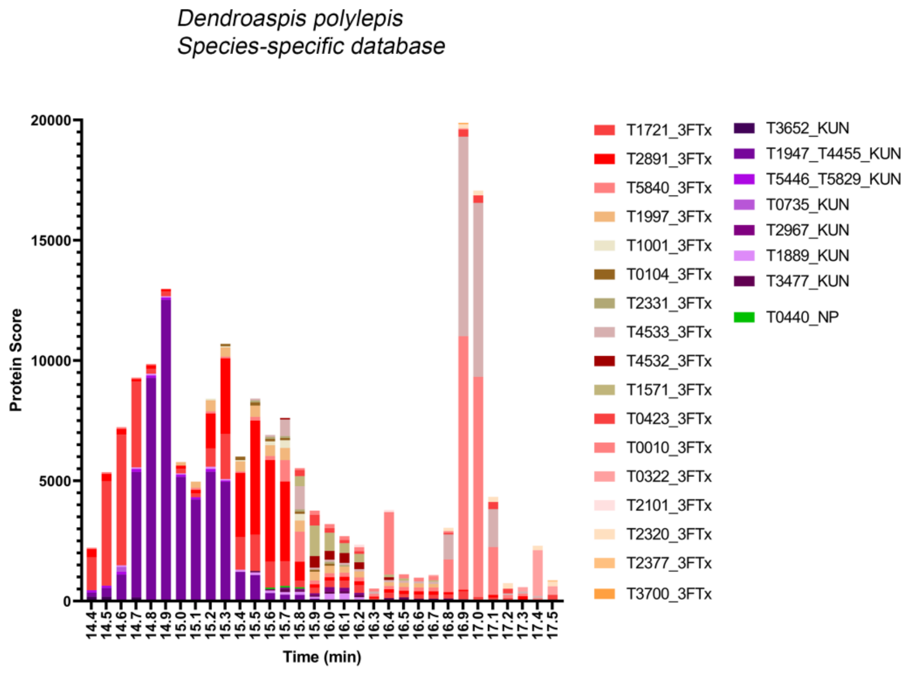

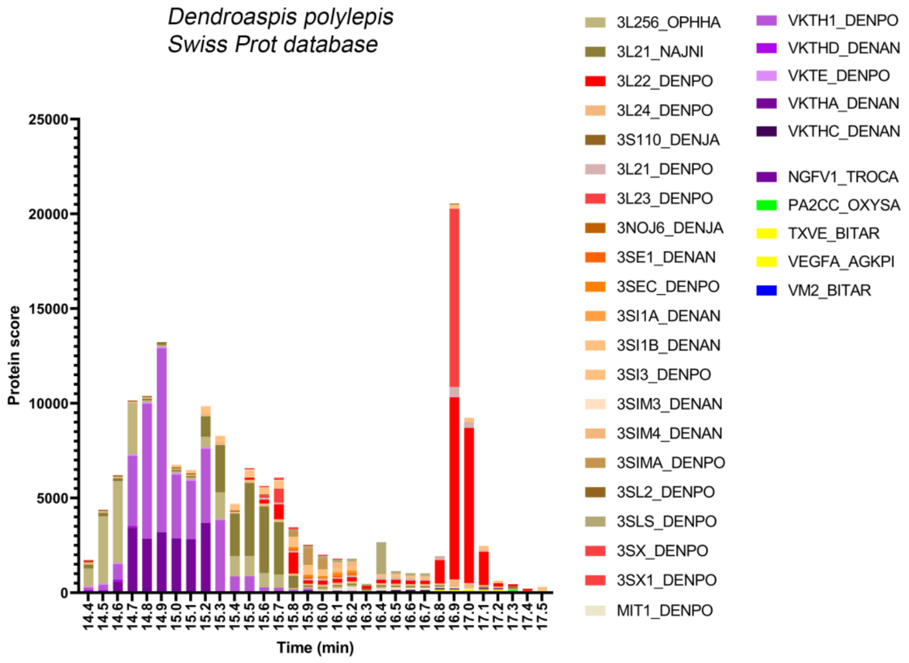

Supplementary Figure S12. Detailed results of proteomics searches using Mascot software against the Species-specific database and Swiss-Prot database to identify anticoagulant venom proteins of *Dendroaspis polylepis*. The protein score represents the probability that designated proteins are present in the sample. 3S/3L/3N/MIT/3FTx = Three-finger toxins, PA2/NP = Neutral phospholipase A_2, VKT/KUN = Kunitz-type serine protease inhibitor, NGFV = venom nerve growth factor, VEGFA = vascular endothelial growth factor, VM = snake venom metalloprotease.

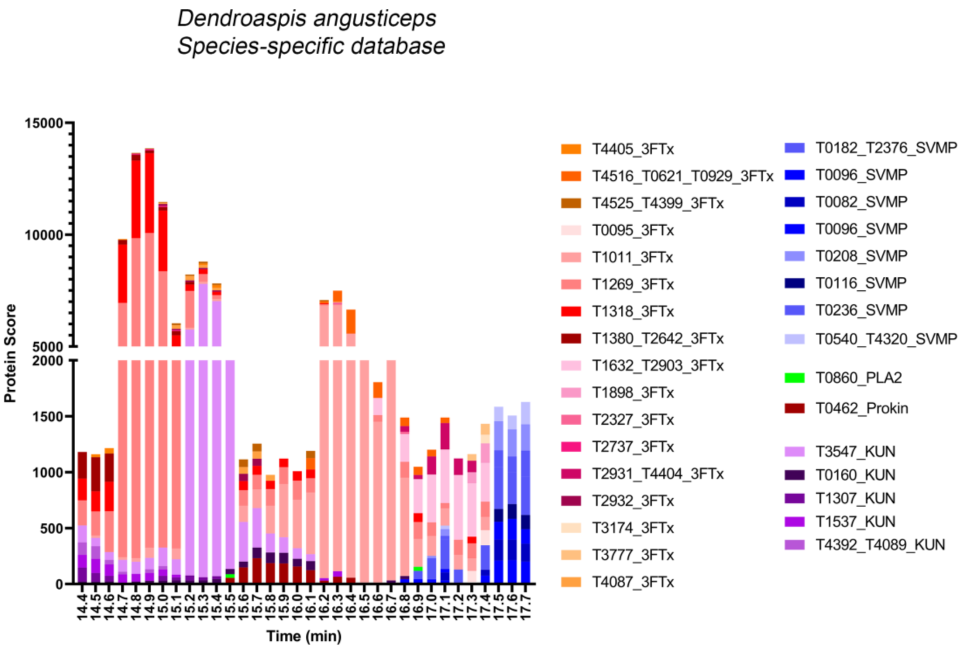

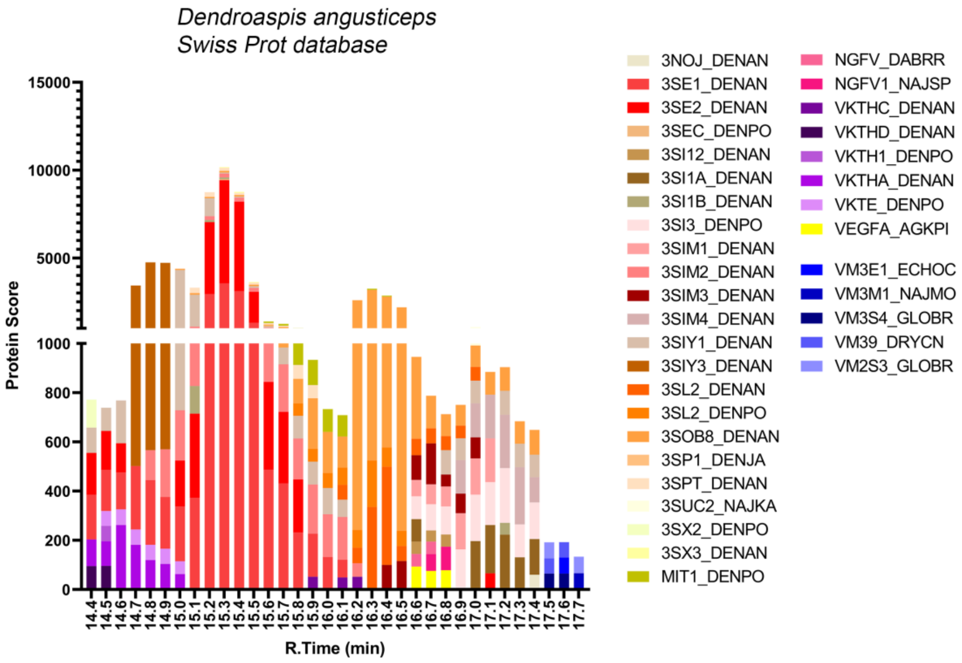

Supplementary Figure S13. Detailed results of proteomics searches using Mascot software against the Species-specific database and Swiss-Prot database to identify anticoagulant venom proteins of *Dendroaspis angusticeps*. The protein score represents the probability that designated proteins are present in the sample. 3S/3N/MIT/3FTx = Three-finger toxins, PLA2 = Phospholipase A_2, VKT/KUN = Kunitz-type serine protease inhibitor, NGFV = venom nerve growth factor, VEGFA = vascular endothelial growth factor, VM/SVMP = snake venom metalloproteinase, Prokin = protein kinase.

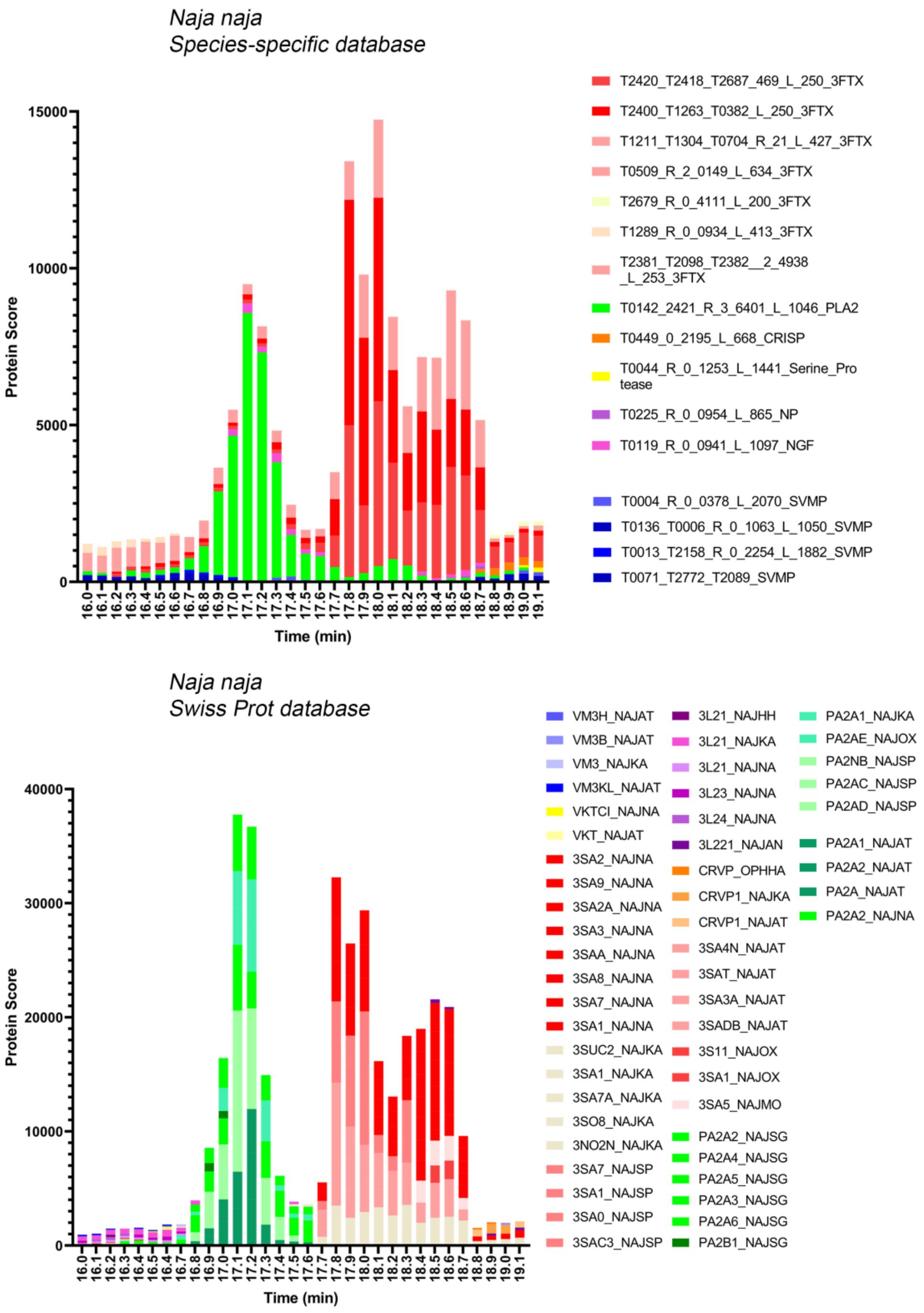

Supplementary Figure S14. Detailed results of proteomics searches using Mascot software against the Species-specific database and Swiss-Prot database to identify anticoagulant venom proteins of *Naja naja*. The protein score represents the probability that designated proteins are present in the sample. 3S/3L/3N/3FTx = Three-finger toxins, PLA2 = Phospholipase A_2, VKT/KUN = Kunitz-type serine protease inhibitor, NGF = venom nerve growth factor, VM/SVMP = snake venom metalloproteinase, CRVP/CRISP = Cysteine-rich venom secretory proteins.

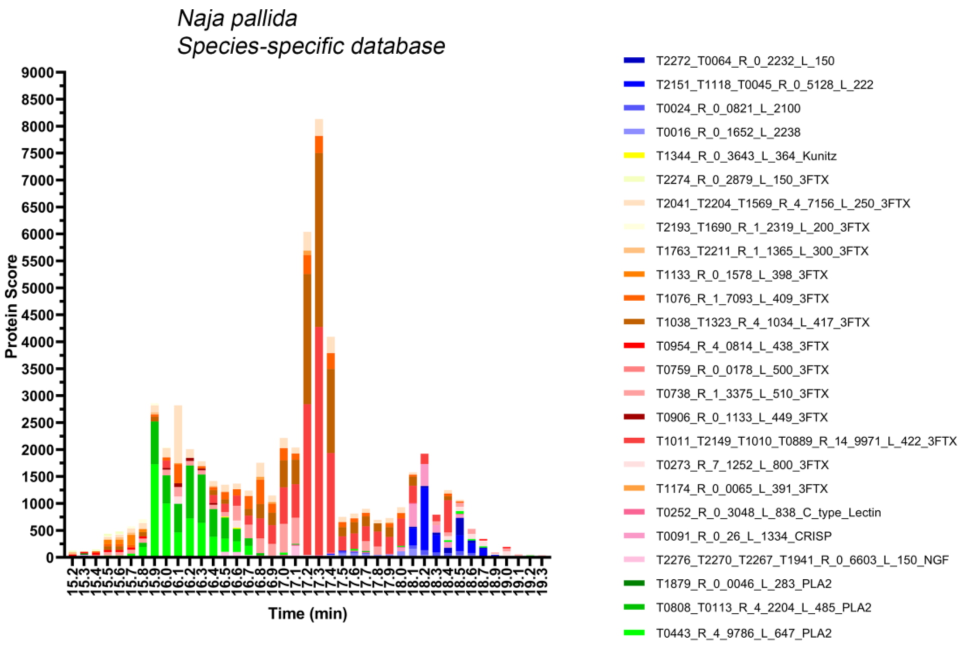

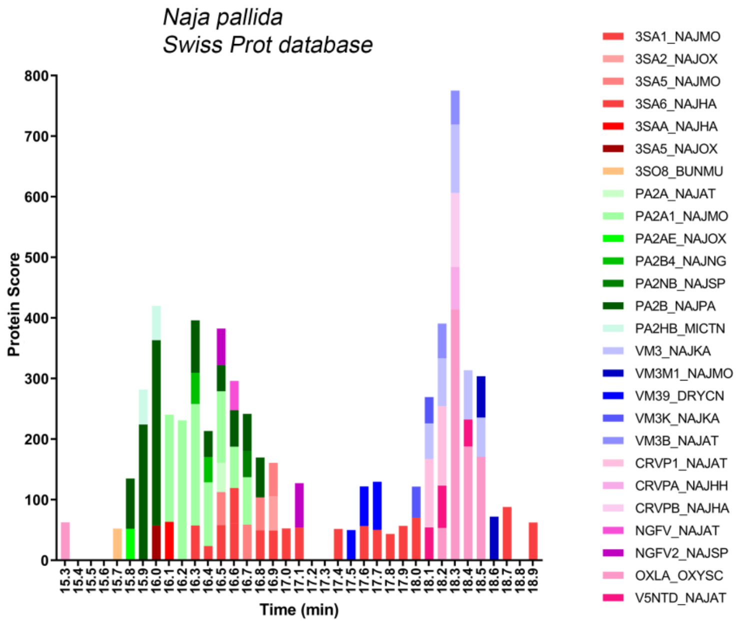

Supplementary Figure S15. Detailed results of proteomics searches using Mascot software against the Species-specific database and Swiss-Prot database to identify anticoagulant venom proteins of *Naja pallida*. The protein score represents the probability that designated proteins are present in the sample. 3S/3FTx = Three-finger toxins, PA/PLA2 = Phospholipase A_2, VKT/KUN = Kunitz-type serine protease inhibitor, NGF = venom nerve growth factor, VM/SVMP = snake venom metalloproteinase, CRVP/CRISP = Cysteine-rich venom secretory proteins, OXLA = L-amino-acid oxidase.

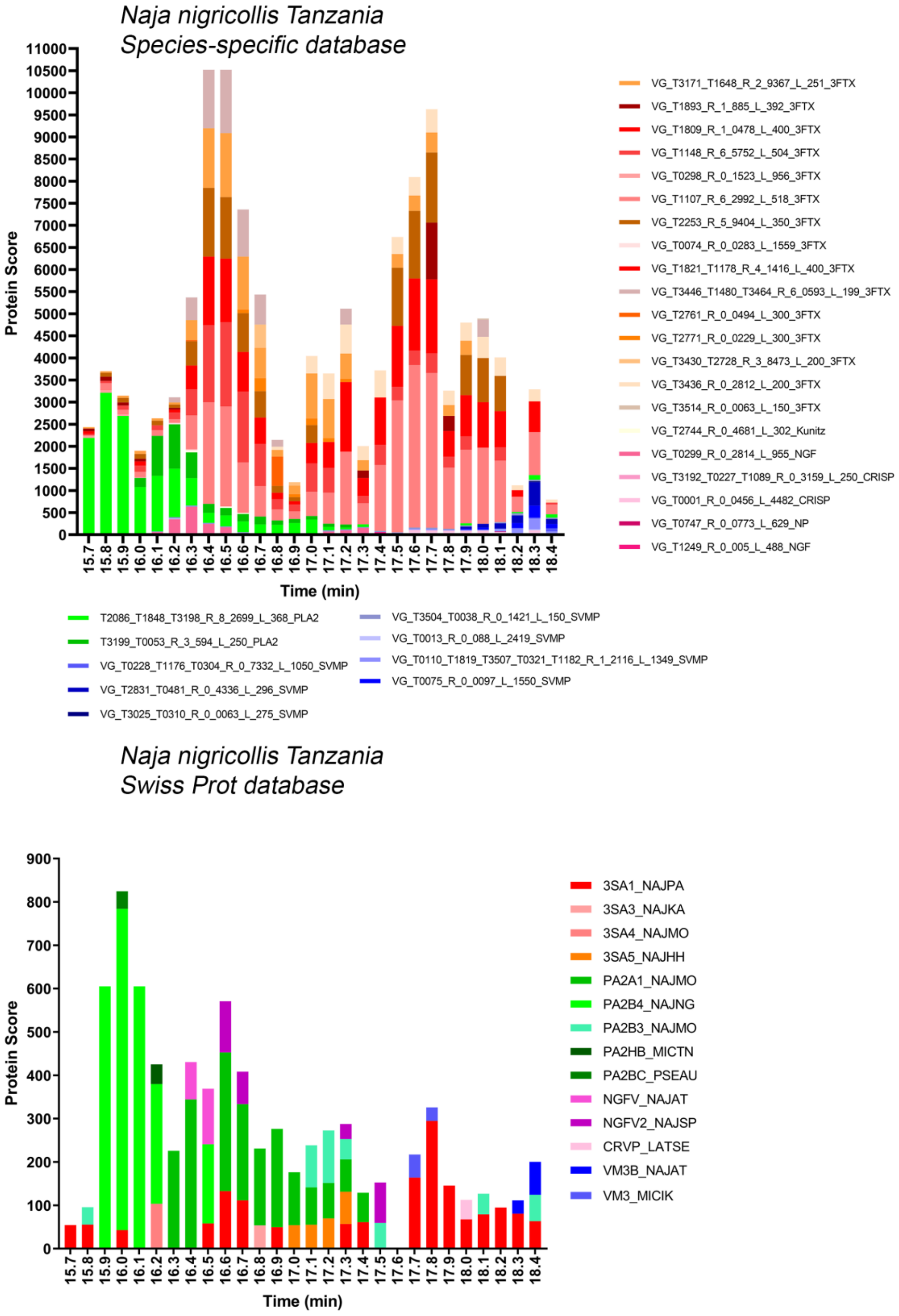

Supplementary Figure S16. Detailed results of proteomics searches using Mascot software against the Species-specific database and Swiss-Prot database to identify anticoagulant venom proteins of *Naja nigricollis Tanzania*. The protein score represents the probability that designated proteins are present in the sample. 3S/3FTx = Three-finger toxins, PA/PLA2 = Phospholipase A_2, VKT/KUN = Kunitz-type serine protease inhibitor, NGF = venom nerve growth factor, VM/SVMP = snake venom metalloproteinase, CRVP/CRISP = Cysteine-rich venom secretory proteins.

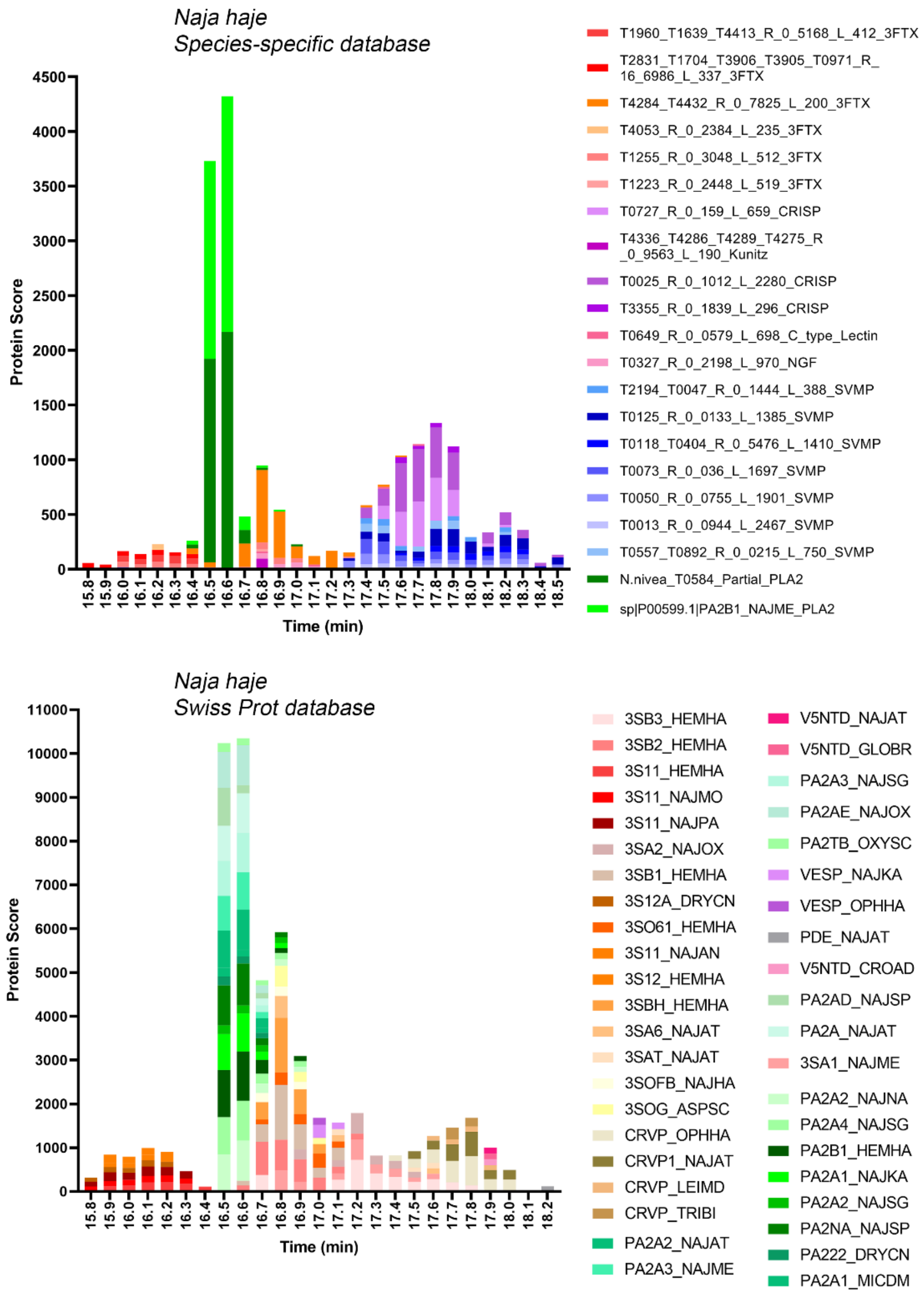

Supplementary Figure S17. Detailed results of proteomics searches using Mascot software against the Species-specific database and Swiss-Prot database to identify anticoagulant venom proteins of *Naja haje*. The protein score represents the probability that designated proteins are present in the sample. 3S/3FTx = Three-finger toxins, PA/PLA2 = Phospholipase A_2, VKT/KUN = Kunitz-type serine protease inhibitor, NGF = venom nerve growth factor, VM/SVMP = snake venom metalloproteinase, CRVP/CRISP = Cysteine-rich venom secretory proteins.

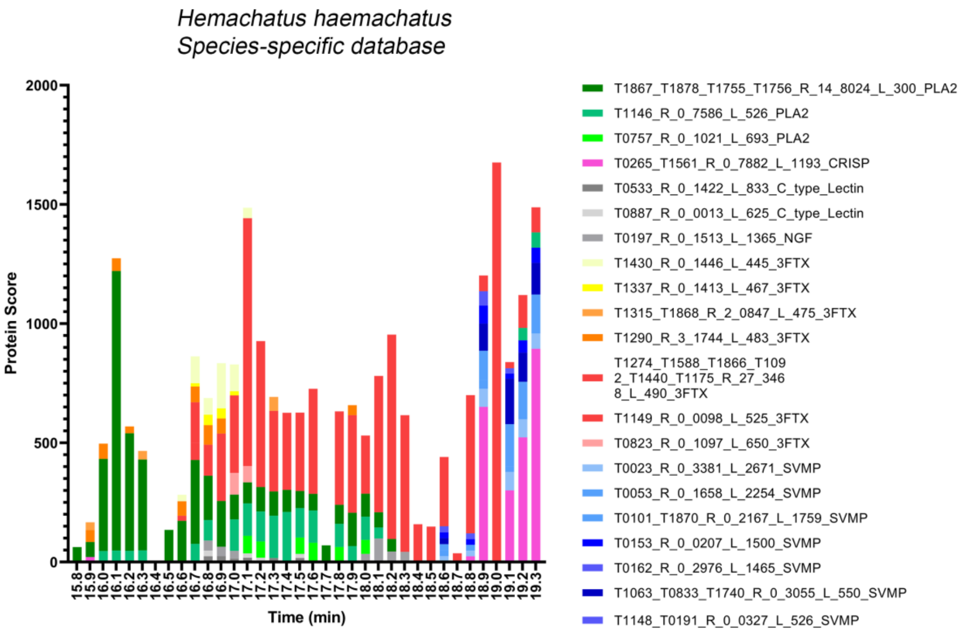

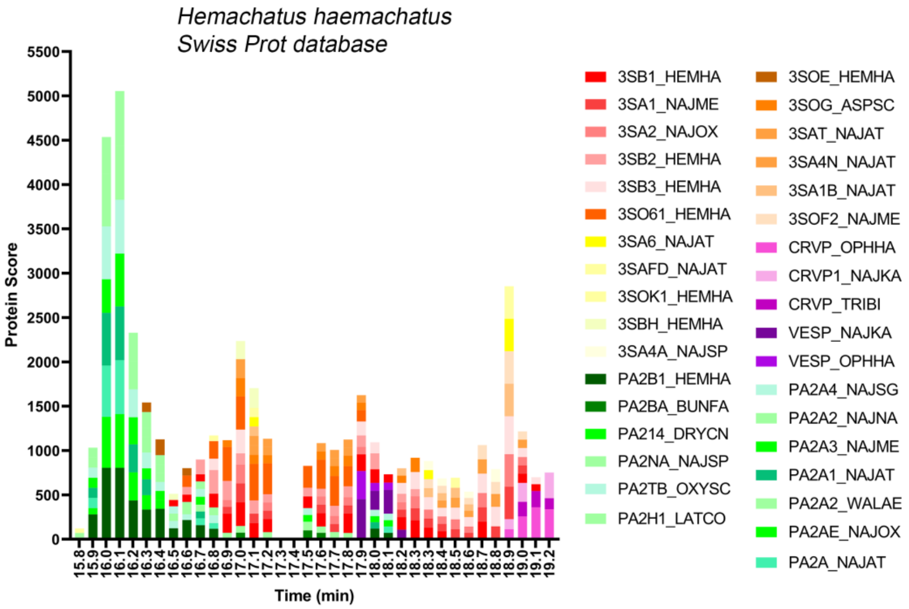

Supplementary Figure S18. Detailed results of proteomics searches using Mascot software against the Species-specific database and Swiss-Prot database to identify anticoagulant venom proteins of *Naja haje*. The protein score represents the probability that designated proteins are present in the sample. 3S/3FTx = Three-finger toxins, PA/PLA2 = Phospholipase A_2, NGF = venom nerve growth factor, VM/SVMP = snake venom metalloproteinase, CRVP/CRISP = Cysteine-rich venom secretory proteins.
